# Modeling the dynamics of hepatic metabolism: the predominance of 12-hour rhythmicity in metabolic adaptation

**DOI:** 10.1007/s00018-025-06046-4

**Published:** 2026-01-09

**Authors:** Madlen Matz-Soja, Christiane Körner, Fritzi Ott, Janett Fischer, Eugenia Marbach-Breitrück, Christian Bergmann, Ute Hofmann, Andrej Shevchenko, Iwona Wallach, Kathrin Textoris-Taube, Michael Mülleder, Rolf Gebhardt, Thomas Berg, Nikolaus Berndt

**Affiliations:** 1 Division of Hepatology, Department of Medicine II, Laboratory for Clinical and Experimental Hepatology, Leipzig University Medical Center, Leipzig, Germany; 2 Rudolf-Schönheimer-Institute of Biochemistry, Faculty of Medicine, Leipzig University, Leipzig, Germany; 3 Dr. Margarete Fischer‑Bosch Institute of Clinical Pharmacology, University of Tübingen, Stuttgart, Germany; 4 Max Planck Institute of Molecular Cell Biology and Genetics, Dresden, Germany; 5 Department of Radiology, , Charité – Universitätsmedizin Berlin, corporate member of Freie Universität Berlin and Humboldt-Universität zu Berlin, Berlin, Germany; 6 Core Facility High Throughput Mass Spectrometry, Charité - Universitätsmedizin Berlin, corporate member of Freie Universität Berlin and Humboldt-Universität zu Berlin, Berlin, Germany; 7 Department of Molecular Toxicology, German Institute of Human Nutrition Potsdam-Rehbruecke (DIfE), Nuthetal, Germany

**Keywords:** Circadian/diurnal rhythm, Liver, Metabolism, Mitochondria, Kinetic modeling, Multiomics, HEPATOKIN1

## Abstract

**Background and objectives:**

The liver continuously adjusts its metabolic activity to synchronize the nutrient supply with the body’s demands. This synchronization involves the complex coordination of acute metabolic needs, nutrient availability, and activity levels, which is orchestrated according to cyclic internal rhythms governed by the circadian clock. This study aimed to decipher the role of circadian rhythms in liver metabolic functions, including mitochondrial activities that are critical for energy production and metabolic adaptation.

**Methods:**

We investigated rhythmic changes in liver metabolism via comprehensive multiomics and kinetic mathematical modeling. The liver proteome of male mice was analyzed and modeled, and complementary serum lipidomic and metabolomic analyses were performed. Mitochondrial proteins were examined to evaluate the role of mitochondria in the oscillating regulation of energy production.

**Results:**

Most metabolic functions, particularly those related to carbohydrate and fatty acid metabolism, exhibit rhythmic patterns on a 12-hour rather than a 24-hour cycle. The importance of this rhythmicity is function-dependent and can account for 25% to 50% of the overall variability. Mitochondrial activities also exhibit temporal fluctuations that are closely linked to nutrient availability. The strong correlation between metabolic functions and serum metabolites highlights the precise alignment between physiological demand and metabolic performance.

**Conclusions:**

Hepatic metabolic functions follow a 12-hour cycle rather than a 24-hour cycle, significantly contributing to the liver’s ability to meet nutrient demands throughout the day. Mitochondrial dynamics, which are influenced by nutrient availability, play a central role in adapting energy production to the body’s metabolic needs.

**Supplementary Information:**

The online version contains supplementary material available at https://doi.org/10.1007/s00018-025-06046-4.

## Introduction

The liver is a central metabolic hub, overseeing vital physiological functions that are crucial for maintaining homeostasis. Activities of the liver are governed by the availability of nutrients and hormones, as well as by systemic metabolic demands. As expected, these metabolic activities align closely with diurnal cycles, driven by central clock genes that regulate circadian rhythms through complex feedback loops involving key transcription factors such as ARNTL/BMAL1 and CLOCK [1, 2]. The brain’s master clock, located in the suprachiasmatic nucleus, induces peripheral tissues, including the liver, to synchronize their oscillations with the body’s overall circadian rhythm [3]. External factors such as sleep patterns and dietary habits further shape the circadian regulation of the liver, highlighting the role of the liver as not only a metabolic powerhouse but also a finely tuned orchestrator of physiological rhythms [4, 5].

In addition to the central role of the liver in metabolism, distinct circadian fluctuations in mitochondrial function have been observed. Mitochondrial processes, such as oxidative phosphorylation (OXPHOS), exhibit robust daily rhythms that are closely linked to the energy requirements of the cell. Mitochondrial activity is adapted to align with diurnal variations in substrate utilization and respiration, thereby optimizing energy production and oxidative metabolism in liver cells. These rhythms are not only coordinated by the cellular clock but also influenced by mitochondria-specific regulators, which indicates the unique temporal control of mitochondrial function compared with overall cellular energy management [6].

Recent studies have highlighted the critical role of nutrient availability in modulating the circadian regulation of liver metabolism, distinguishing the regulation of the liver from regulatory mechanisms in other organs [5, 7, 8]. High-throughput technologies have enabled detailed monitoring of circadian changes in gene and protein abundances, revealing significant variability in enzyme levels throughout the day. Over the past two decades, research has revealed the molecular links between circadian clock genes and liver function, often through studies in genetically modified mice. These investigations have established potential connections between clock dysfunction and liver disorders such as metabolic liver disease, hepatitis, and hepatocellular carcinoma (HCC), with the results suggesting a bidirectional relationship through which liver diseases can also impact circadian function [9].

Despite advancements in identifying circadian changes at the molecular level, understanding their functional implications remains challenging. Traditional bioinformatics and systems biology approaches, such as gene set enrichment and flux balance analysis, provide valuable insights into molecular signatures and metabolic network dynamics [10, 11]. However, these methods often overlook the central role of enzymes and transporters in regulating metabolic states. Kinetic models, known as ‘virtual twins’, offer a mechanistic understanding of these regulatory components, allowing for predictions of metabolic functions under various conditions, from healthy states to models of diseases such as metabolic dysfunction-associated steatohepatitis (MASH) and HCC [12].

In this study, we employed a multiomics approach and analyzed proteomic, metabolomic, and lipidomic data to investigate rhythmic changes in liver metabolism. Using the HEPATOKIN1 mathematical model, we examined the functional consequences of these rhythms [13]. Our findings reveal that despite asynchronous rhythms of individual enzymes in transcriptomic and proteomic data, many metabolic functions exhibit 12-hour rhythms, challenging the conventional focus on a 24-hour cycle. The correlation between serum metabolite levels and metabolic functions underscores the precise coordination required to meet physiological demands, suggesting that the 12-hour period plays an important role in hepatic metabolism and that a reevaluation of the temporal dynamics that govern liver function is necessary.

## Materials and methods

### Study design and animal care

Male C57BL/6 N mice were housed in a pathogen-free facility on a 12:12-hour light-dark cycle (lights on at 6 a.m. = Zeitgeber Time (ZT)0, lights off at 6 p.m. = ZT12) in a cage of max. 3 animals in accordance with national guidelines and the World Medical Association Declaration of Helsinki. The animal experiments were approved locally (permission numbers: T04/14 and N03/14). Mice had free access to regular chow (ssniff^®^ R/M-H; 24.0 kJ% protein, 67 kJ% carbohydrate, 9 kJ% fat) and tap water. At 12 weeks, mice were euthanized at ZT0, ZT3, ZT6, ZT9, ZT12, ZT16, and ZT20 after anesthesia with ketamine, xylazine, and atropine. Blood was collected from the beating hearts without coagulants. After letting the samples clot for 10 min at room temperature, they were centrifuged at 2000 x g for 10 min. The resulting serum was snap-frozen for metabolome and lipidome analyses. Liver tissue was snap-frozen for proteome analyses.

Sample size for each group was determined based on preliminary data and practical considerations. Pilot studies informed expected variability and effect sizes, suggesting 3–5 animals per group to detect meaningful differences without excessive variability. Ethical and logistical factors were also considered, aiming to reduce animal use while ensuring robust results. The inclusion criteria were 12 weeks of age and healthy status. Unwell animals were excluded.

### Shotgun proteome profiling and data analysis

Liver tissue samples isolated at ZT0, ZT3, ZT6, ZT9, ZT12, ZT16, and ZT20 were analyzed for protein abundance (biological replicates: *N* = 3–5, technical replicates *n* = 2). LC‒MS/MS was carried out via nanoflow reverse-phase liquid chromatography with a Dionex Ultimate 3000 chromatograph (Thermo Fisher Scientific, Waltham, MA) coupled online to a Q-ExactivePlus Orbitrap mass spectrometer (Thermo Fisher Scientific). The samples were analyzed via a two-linear-column system. For detailed information, see the Supplementary Methods (Online Resource 1) and Table S1 (Online Resource 2).

### Lipidome analysis

Lipidome analysis was performed using a modified protocol based on Folch et al. (1957) and analyzed via shotgun mass spectrometry [14]. Briefly, lipids were extracted from liver tissue, prepared with internal standards, and analyzed in both positive and negative ion modes using a Q-Exactive mass spectrometer. The biological replicates in lipidome analysis were *N* = 3, the technical replicates *n* = 2. For detailed information, see the Supplementary Methods (Online Resource 1) and Table S2 (Online Resource 3).

### Metabolome analysis

Metabolome analysis was conducted to determine the levels of amino acids, urea, bile acids, and other metabolites in serum via GC-MS and LC-MS/MS methods [15, 16]. Bile acids were analyzed using an Agilent 6460 triple quadrupole mass spectrometer with multiple reaction monitoring (MRM) mode. The biological replicates in metabolome analysis were *N* = 3, the technical replicates *n* = 1. Detailed procedures and statistical analyses are described in the Supplementary Methods (Online Resource 1) and Table S3 (Online Resource 4).

### Glycogen and triglyceride (TAG) assays

The glycogen content in liver tissues (ZT0, ZT3, ZT6, ZT9, ZT12, ZT16, and ZT20) was quantified using an Abcam Glycogen Assay Kit (ab65620, Abcam, Cambridge, UK) according to the manufacturer’s instructions for tissue samples less than 100 mg. Defined amounts of liver tissue were homogenized in 200 µL ddH2O using a Precellys^®^ 24 (Bertin, Montigny-le-Bretonneux, France) to isolate glycogen as described in the instructions. For each sample, 3 µL was diluted to fit the standard curve range. Optical density (OD) was measured at 570 nm with a SpectraMax^®^ iD5 (Molecular Devices, San Jose, CA, USA).

The TAG content in liver tissue was quantified using an Abcam Triglyceride Assay Kit (ab65336, Abcam) according to the manufacturer’s instructions. Defined amounts of liver tissue were homogenized in 1 mL of 5% NP-40/ddH2O solution using a Precellys^®^ 24 to isolate TAGs as described in the instructions. Undiluted samples were measured with a fluorometric assay at Ex/Em = 535/587 nm using a SpectraMax^®^ iD5.

The results were normalized to the amount of liver tissue used in the experiment. The biological replicates in both assays were *N* = 3–5, the technical replicates *n* = 2.

### Assessment of metabolic capacities

Metabolic capacities were assessed using the HEPATOKIN1 [13] model coupled with a detailed lipid droplet metabolism model [17] as described previously [18]. This model integrates major hepatic pathways and mitochondrial processes, coupled with hormonal signaling for insulin and glucagon. Individual model instantiations for each sample were generated using protein intensity profiles, with glucose concentrations ranging from fasting to well-fed states.

The model integrates central biochemical pathways including glycogen metabolism, fructose metabolism, galactose metabolism, glycolysis, gluconeogenesis, the oxidative and non-oxidative branches of the pentose phosphate pathway, fatty acid synthesis, TAG synthesis, lipid droplet formation and degradation, very low-density lipoprotein (VLDL) synthesis, cholesterol synthesis, the tricarboxylic acid cycle, the respiratory chain, oxidative phosphorylation, β-oxidation of fatty acids, the urea cycle, ethanol metabolism, ketone body synthesis, and amino acid metabolism. Each enzymatic step is described by a kinetic rate law that captures the specific enzymatic kinetics of hepatocytes, including regulation through substrate availability, allosteric effectors, and – where relevant – the phosphorylation state of interconvertible enzymes. Hormone-dependent regulation (e.g., insulin/glucagon) of liver metabolism via reversible enzyme phosphorylation is incorporated through a phenomenological transfer function, as described by Berndt et al. [19]. The resulting system of ordinary differential equations describes the time course of metabolite concentrations and metabolic fluxes based on the kinetic parameters of the enzymes, substrate availability, and protein levels.

Sample-specific model instantiations were generated based on individual proteomic profiles by scaling each enzyme’s V_max_ according to its measured proteomic abundance, assuming proportionality between protein amount and catalytic capacity [20]. For each sample, metabolic capacities were assessed by calculating maximal fluxes under predefined metabolic conditions as described by Berndt et al. [13]. Metabolic functions under physiological conditions were assessed under the assumption that plasma metabolite concentrations are not independent of each other. Under physiological conditions, glucose stimulates insulin release from beta cells, concomitantly reducing glucagon release from alpha cells in the pancreas, and both hormones control the release of fatty acids from adipose tissue. The interdependence between plasma glucose, plasma hormones, and plasma fatty acid concentrations was considered using the sigmoid Hill-type function, which experimentally determines the glucose-insulin, glucose-glucagon, and glucose-fatty acid relations [13, 17, 19]. Variations of plasma glucose levels were considered, ranging from a fasted state to excessive nutrient uptake (3–12 mM plasma glucose). This approach enables the sample-specific assessment of metabolic capacities under standardized conditions. The only difference between the samples lies in the abundance of the metabolic enzymes, and any change in metabolic *capacities* results purely as a consequence of protein rhythmicity. For a detailed description, refer to the Supplementary Methods and Fig. S1 (Online Resource 1) and Table S4 (Online Resource 5). Fig. S1 shows a schematic overview of the key modeled pathways, illustrating the coverage of central carbon metabolism, lipid metabolism, and mitochondrial processes in HEPATOKIN1. Table S4 summarizes the analyzed metabolic functions – classified as either within the physiological range or at maximal capacity – and lists all significantly associated proteins along with their corresponding *p*-values.

### Mitochondrial model description

The kinetic mitochondrial model includes major metabolic pathways of mitochondrial energy metabolism from pyruvate and fatty acids. It also incorporates key electrophysiological processes of the inner mitochondrial membrane and uses first-order differential equations to describe time-dependent variations in metabolites and ions. Detailed model parameters and evaluation methods are provided in the Supplementary Methods (Online Resource 1).

### BioDare2 analysis

The rhythmicity of the proteome, metabolome, and lipidome was assessed using the BioDare2 online platform [21], which is designed for circadian and other time-series data analysis. Only molecular features (proteins, metabolites, and lipid classes) detected across all seven ZT points were included. BioDare2 fits each time-series trace to a library of sine-wave templates, estimating the period, phase (peak time), and amplitude, and calculates empirical p-values using 10,000 null permutations. We applied both available algorithms – BD2eJTK and eJTKClassic – over a period range of 8–28 h (with default detrending and no smoothing). For each feature, the test yielding the more significant result was retained. P-values were adjusted using the Benjamini-Hochberg method (q < 0.05), and peak phases were extracted for further visualization and statistical analysis. Additionally, mitochondrial-specific proteins were identified from the liver proteomics dataset using the Mouse MitoCarta 3.0 database [22], and their rhythmicity was analyzed using the same BioDare2 pipeline.

### Visualization and statistical evaluation

The data were plotted as the means of biological replicates (*n* = 3–5) with either the range or standard deviation (as indicated in the figure legends). For visualization, the values for time point ZT0 were cloned and recorded as the final value (ZT24) in each diagram to complete one diurnal cycle; these cloned values were not considered in any calculation.

To evaluate rhythmicity in protein expression and metabolic functions, we tested three models: (i) a non-rhythmic constant model (mean value), (ii) a 12-hour sinusoidal model, and (iii) a 24-hour sinusoidal model. Each sinusoidal fit was constrained to a fixed amplitude (equal to the observed standard deviation), while allowing the phase to vary for optimal fit. Best-fit wave functions with fixed frequencies to 12–24 h were computed, and the fitting parameter was the time shift of the maximal and minimal values. For each model, we calculated the sum of squared deviations (SSD) from the observed data and determined the percent variance explained by each rhythmic model relative to the constant baseline. The fitting results of the comparative model performance are given in Table S5 (Online Resource 6).

The variability of the measured data over the time course was analyzed via one-way ANOVA. The null hypothesis was rejected at the *p < 0.05, **p < 0.01, and ***p < 0.001 levels. Statistical analysis was performed with GraphPad Prism 10.

## Results

### Diurnal rhythmicity of multiomics data reveals a high impact on metabolic pathways

**Fig. 1:**
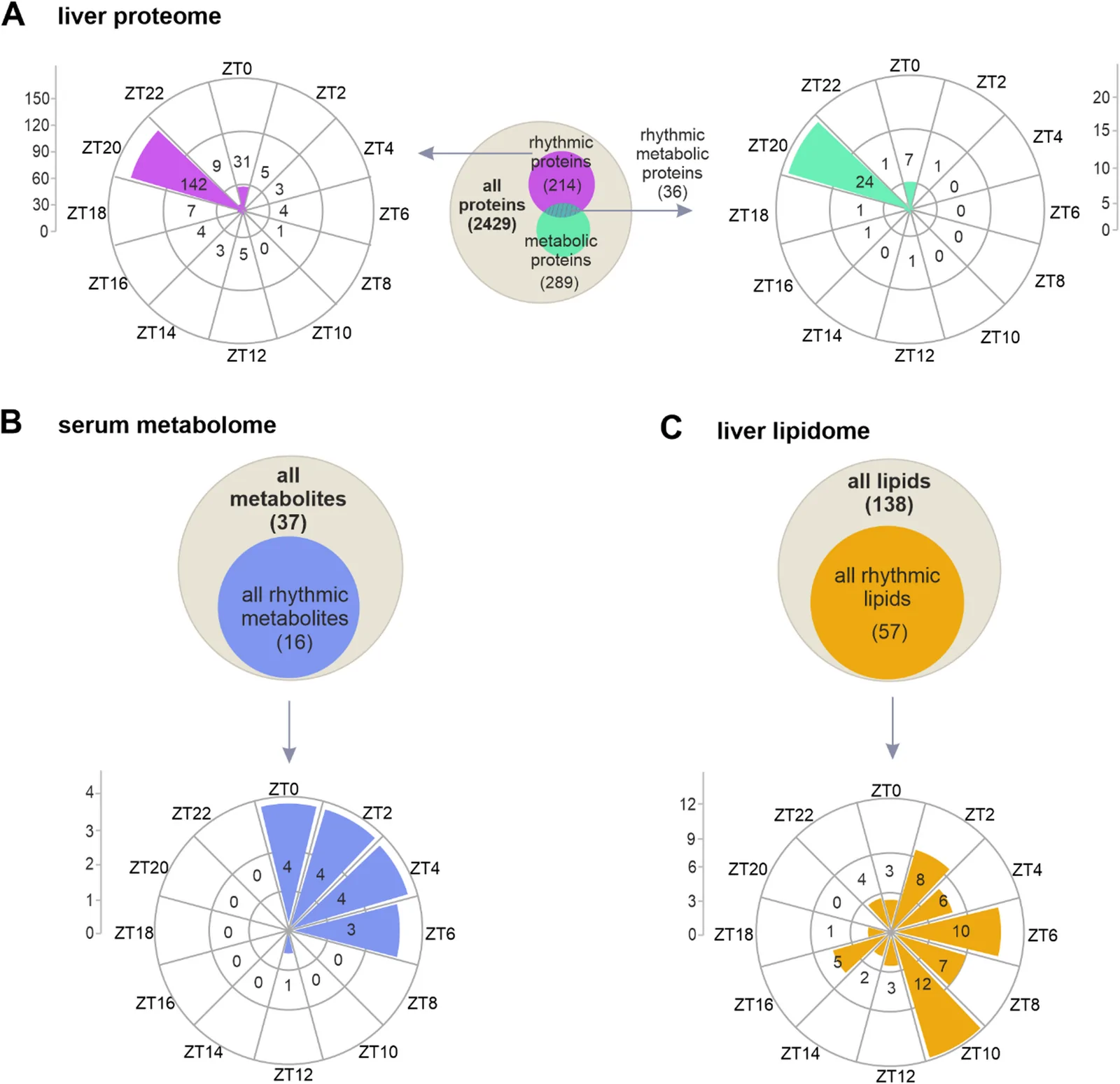
Rhythmicity analysis of proteome, metabolome, and lipidome datasets. Datasets were analyzed for rhythmicity using the BioDare2 online tool. **A** Liver proteome: Venn diagram indicates the number of rhythmic proteins (*n* = 214) and the subset involved in metabolic processes (*n* = 289) out of the total quantified proteins (*n* = 2429). Radar plots show the number of significantly rhythmic proteins and significant rhythmic detected metabolic proteins (*n* = 36) peaking at each ZT. **B** Serum metabolome: Out of 37 quantified metabolites, 23 showed significant rhythmicity. The radar plot displays the number of rhythmic metabolites peaking at each ZT. **C** Liver lipidome: Of 138 detected lipids, 75 exhibited rhythmic behavior. The radar plot illustrates the number of rhythmic lipids with peak abundance at the respective ZT time points

The collected datasets, including proteome, metabolome, and lipidome data, were analyzed with the BioDare2 online tool to assess their rhythmicity [21] (Fig. 1A-C; Table S6 in Online Resource 7). Analysis of 2,429 proteins revealed that 8.8% of the proteins exhibited a significant rhythmic expression, peaking at ZT0 (31 proteins) and ZT20 (142 proteins). Notably, 12.4% of the metabolic proteins exhibited a significant rhythmic expression with the same peak times at ZT0 (7 proteins) and ZT 20 (24 proteins) (Fig. 1A). Approximately 43% of the serum metabolites, particularly amino acids such as threonine and tryptophan and free fatty acids such as arachidonic acid (20:4 n-6), exhibited significant rhythmic patterns, with peak concentrations occurring at the beginning of the light phase (Fig. 1B). In the lipidome analysis, 41% (57/138) of the lipid classes in the liver exhibited a significant rhythmic signature. The majority of these lipid classes are integral components of cellular membranes, including phosphatidylcholine (PC), PC ether, lysophosphatidylethanolamine, lysophosphatidylcholine, and phosphatidylinositol. These lipid classes also exhibited peak levels predominantly during the light phase between ZT0 and ZT12 (Fig. 1C).

To explore the temporal interplay between hepatic lipid species and central metabolic pathways, we performed Pearson correlation analyses across six ZT points (ZT0–ZT16) (Fig. S2A-E in Online Resource 1; Table S7 in Online Resource 8). The results reveal dynamic, phase-specific association patterns, supporting a rhythmic coordination between lipid metabolism and core metabolic processes.

TAG species (Fig. S2A) showed the strongest correlations with glycolytic intermediates and amino acids, particularly at ZT6 and ZT9, suggesting active lipogenesis from carbohydrate and amino acid substrates during the light phase. These associations diminished toward ZT12 and ZT16, indicating reduced metabolic coupling at later time points. Diglycerides displayed peak correlations at ZT9 and ZT12 with glutamine, alanine, and nucleotide metabolites, pointing toward a temporally restricted role in anabolic signaling or membrane precursor supply during the light–dark transition (Fig. S2B). Phospholipids, including PCs, phosphatidylethanolamines, and their ether-linked forms (Fig. S2C), were strongly linked to tricarboxylic acid cycle intermediates and nucleotide metabolism at ZT3 and ZT12. These associations may reflect rhythmic membrane remodeling or mitochondrial activity aligned with feeding–fasting transitions. Lysophospholipids (Fig. S2D) showed weaker and more variable patterns, but transient correlations at ZT3 and ZT6 suggest potential involvement in early light-phase lipid signaling or mitochondrial function. Finally, complex lipids such as cholesterol esters, sphingomyelins, ceramides, and phosphatidylinositols (Fig. S2E) were associated with purine metabolites and acylcarnitines, especially at ZT6–ZT12, indicating rhythmic participation in signaling and membrane turnover during the active phase.

These findings highlight that lipid–metabolite interactions in the liver are temporally organized, likely reflecting the integration of circadian and ultradian regulatory programs that orchestrate hepatic metabolic flexibility across the day.

### Glucose and carbohydrate metabolism

**Fig. 2:**
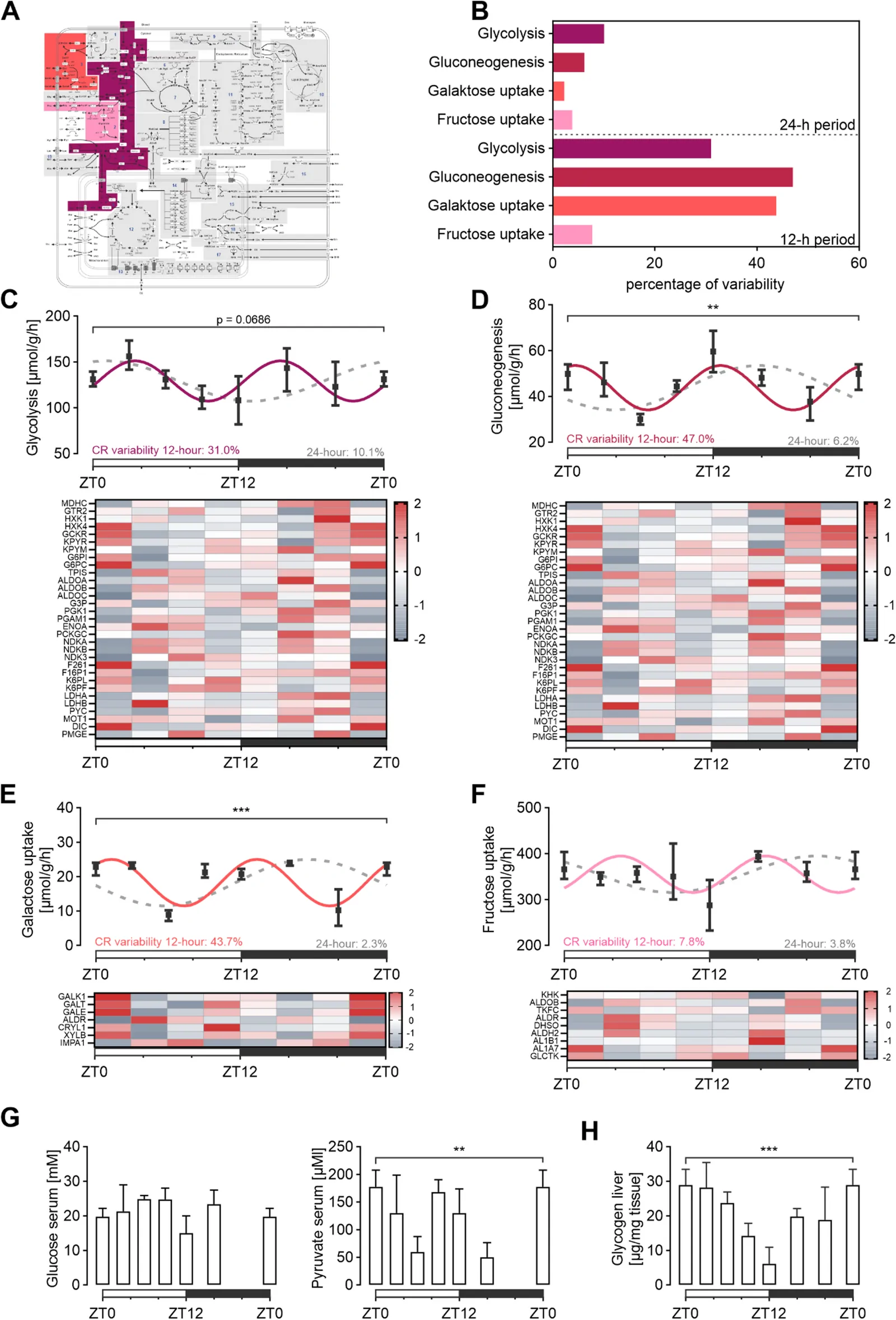
Rhythmic variations of glucose and carbohydrate metabolism. **A** Schematic model representation with colored pathways relevant to carbohydrate metabolism. The scheme was adapted from Berndt et al. [13].​ **B** Relative variability of metabolic functions explained by 12- or 24-hour rhythms. **C**-**F** Maximal calculated capacity of glycolysis, gluconeogenesis, galactose uptake, and fructose uptake. Solid lines indicate best-fit 12-hour sine waves; dashed lines indicate 24-hour rhythms. Below each line graph, a heatmap displays normalized Z-scores of the underlying protein abundances for all enzymes contributing to the respective model pathway. **G** Glucose and pyruvate concentrations detected in serum (*n* = 3). **H** Quantified glycogen content in liver tissue (*n* = 34). (C-H) Values are plotted as mean ± range. One-way ANOVA with *p < 0.05, **p < 0.01, and ***p < 0.001

Glucose homeostasis is a critical hepatic function that maintains plasma glucose levels within the physiological range by balancing glycolysis and gluconeogenesis based on substrate availability [23]. Coregulation of these pathways is essential to prevent futile cycles. Our findings show that both glycolysis and gluconeogenesis followed a strong 12-hour rhythm, contributing 31% and 47%, respectively, to their overall functional variability. In contrast, a 24-hour rhythm accounted for only 10% and 6% of the variability in glycolysis and gluconeogenesis, respectively (Fig. 2A-D).

Glycolysis and gluconeogenesis exhibited asynchronous cycles: gluconeogenic capacity peaked during the day-to-night transition and decreased during nocturnal feeding, while glycolytic capacity was highest during the night when mice were fed. Analysis of diurnal variation in the expression of proteins in these pathways revealed several enzymes significantly associated with their capacities (Fig. S3A-C in Online Resource 1).

Our data demonstrated that glycolytic capacity was negatively correlated with the expression levels of glucose-6-phosphatase (G6PC) and fructose-1,6-bisphosphatase 1 (F16P1), two key regulatory enzymes that inhibit glycolysis, and positively correlated with glucose transporter 2 (GTR2) and pyruvate kinase (KPYM), which increase glycolytic activity (Fig. 2C, Fig. S3A-C). These findings suggest that the 12-hour rhythm of glycolysis is governed by a balance of positive and negative regulatory factors Glycolysis was also positively correlated with lactate dehydrogenase A (LDHA), which indicates increased substrate availability; a positive correlation with phosphoenolpyruvate carboxykinase (PCKGC) and a negative correlation with pyruvate kinase (KPYR) suggested a decrease in the number of futile cycles. Furthermore, glycolysis was positively correlated with pyruvate carboxylase (PYC), which inhibits gluconeogenesis, and negatively associated with 6-phosphofructokinase (K6PL), which promotes gluconeogenesis by suppressing glycolysis (Fig. 2C, Fig. S3B-C). G6PC is an example of coregulation, favoring glucose conversion over fructose-6-phosphate conversion.

We also explored the diurnal variability in the maximal capacities for galactose and fructose utilization. While the 12-hour rhythm of galactose utilization capacity was strong (44%), the variability in fructose utilization capacity was less well explained (Fig. 2E + F; Fig. S4 in Online Resource 1). It remains unclear whether this lack of clarity is due to the absence of a rhythm in this metabolic function or if the data are too noisy to detect it.

Serum glucose (*p* = 0.01233) and pyruvate (*p* = 0.00077) levels in mice exhibited an ultradian rhythm that was correlated with gluconeogenesis (Fig. 2G; Table S8 in Online Resource 1), indicating functional rhythms in serum metabolite concentrations. Additionally, liver glycogen levels decreased during the light phase, reaching a minimum at ZT12, and increased during the dark phase, reflecting the feeding patterns of the mice (Fig. 2H).

### Fatty acid metabolism

**Fig. 3:**
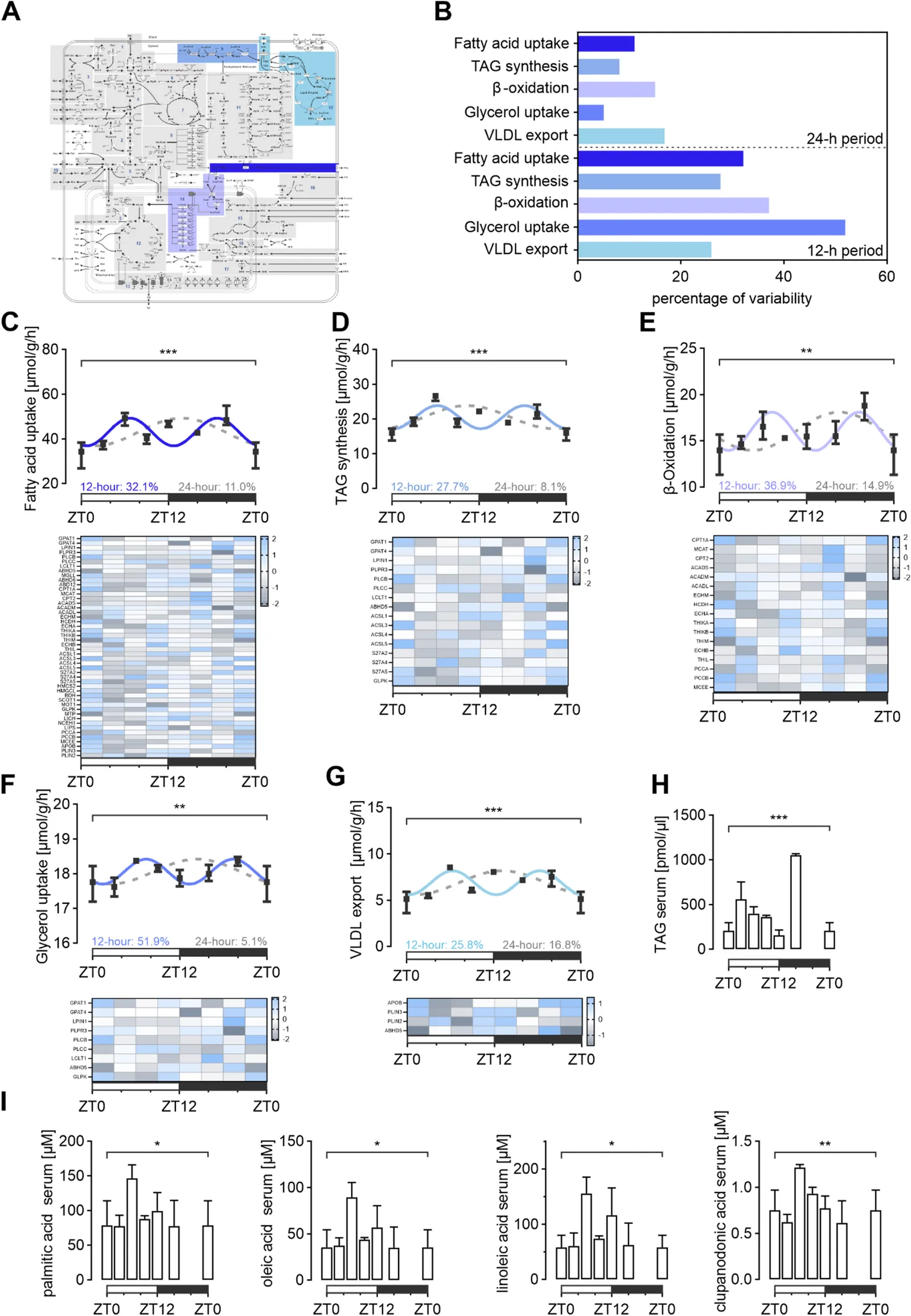
Rhythmic variations of lipid metabolism-related functions I. **A** Schematic model representation with colored pathways relevant to lipid metabolism. **B** Relative variability of metabolic functions explained by 12- or 24-hour rhythms. **C**-**G** Maximal calculated capacity of fatty acid uptake, TAG synthesis, β-oxidation, glycerol uptake, and VLDL export. Solid lines indicate fitted 12-hour sinusoidal patterns, dashed lines indicate 24-hour rhythms. Corresponding heatmaps below each graph show normalized Z-scores of protein abundances used in the respective model modules. **H** TAG content in serum (*n* = 2–3) (**I**) Concentrations of palmitic, oleic, linoleic, and clupanodonic acid detected in serum (*n* = 3). (C-I) Values are plotted as mean ± range. One-way ANOVA with *p < 0.05, **p < 0.01, and ***p < 0.001

Lipids are the primary energy substrates for the liver, with fatty acid uptake, storage, synthesis, and lipoprotein utilization being critical hepatic functions. The metabolic processes of fatty acid uptake, TAG synthesis, and VLDL export showed substantial temporal patterns with 12-hour rhythms, accounting for 32%, 28%, and 26% of the variability, respectively (Fig. 3B-G). As these functions are sequentially linked, their capacities peaked synchronously in the middle of the day and night, with disruption only at ZT12, corresponding to the light-to-dark transition.

Rhythmic changes in fatty acid metabolism were observed in several key proteins (Fig. S5A-E in Online Resource 1). Long-chain fatty acid transport protein/very long-chain acyl-CoA synthetase (S27A2/5) and acyl-CoA synthetase long chain family member 1/3/5 (ACLS1/3/5) exhibited partial ultradian oscillations, peaking at night, reflecting the primary period of fatty acid uptake (Fig. S5A). Similarly, glycerol-3-phosphate acyltransferase 1 (GPAT1), glycerol kinase (GLPK), and perilipin-3 (PLIN3) exhibited robust 12-hour rhythms, peaking in the middle of both light and dark phases. Conversely, lysosomal acid lipase/cholesteryl ester hydrolase (LICH), involved in TAG hydrolysis, peaked during the light phase when TAGs are needed for energy production. Apolipoprotein B-100 (APOB) increased in coordination with lipid-related functions, peaking during the feeding phase (Fig. S5E). The peak in serum TAG levels reflects the predicted increase in VLDL export (Fig. 3H). Phosphatidylethanolamine ether also peaked at night, but other serum lipid classes showed no significant rhythmicity (Fig. S6 in Online Resource 1).

The diurnal patterns of fatty acid metabolic capacity strongly correlated with serum levels of key saturated and unsaturated fatty acids, such as palmitic, oleic, linoleic, and clupanodonic acids (Fig. 3I; Fig. S7 and Tables S9-10 in Online Resource 1). Glycerol uptake, related to fatty acid turnover as the backbone of TAGs, showed a 12-hour rhythm accounting for 52% of its variability, peaking with TAG synthesis in the middle of the day and night.

Depending on cellular energy status, fatty acids undergo β-oxidation to produce acetyl-CoA for ATP or ketone body synthesis. β-oxidation showed 37% functional variability due to a 12-hour rhythm (Fig. 3B, E), with capacity remaining nearly constant between ZT3 and ZT16 and increasing at ZT20. Central enzymes like carnitine palmitoyltransferase 1 A (CPT1A) exhibited this rhythm, while others like enoyl-CoA hydratase (ECHM) and 3-ketoacyl-CoA thiolase (THIM) had opposite patterns (Fig. S5B, D in Online Resource 1).

**Fig. 4:**
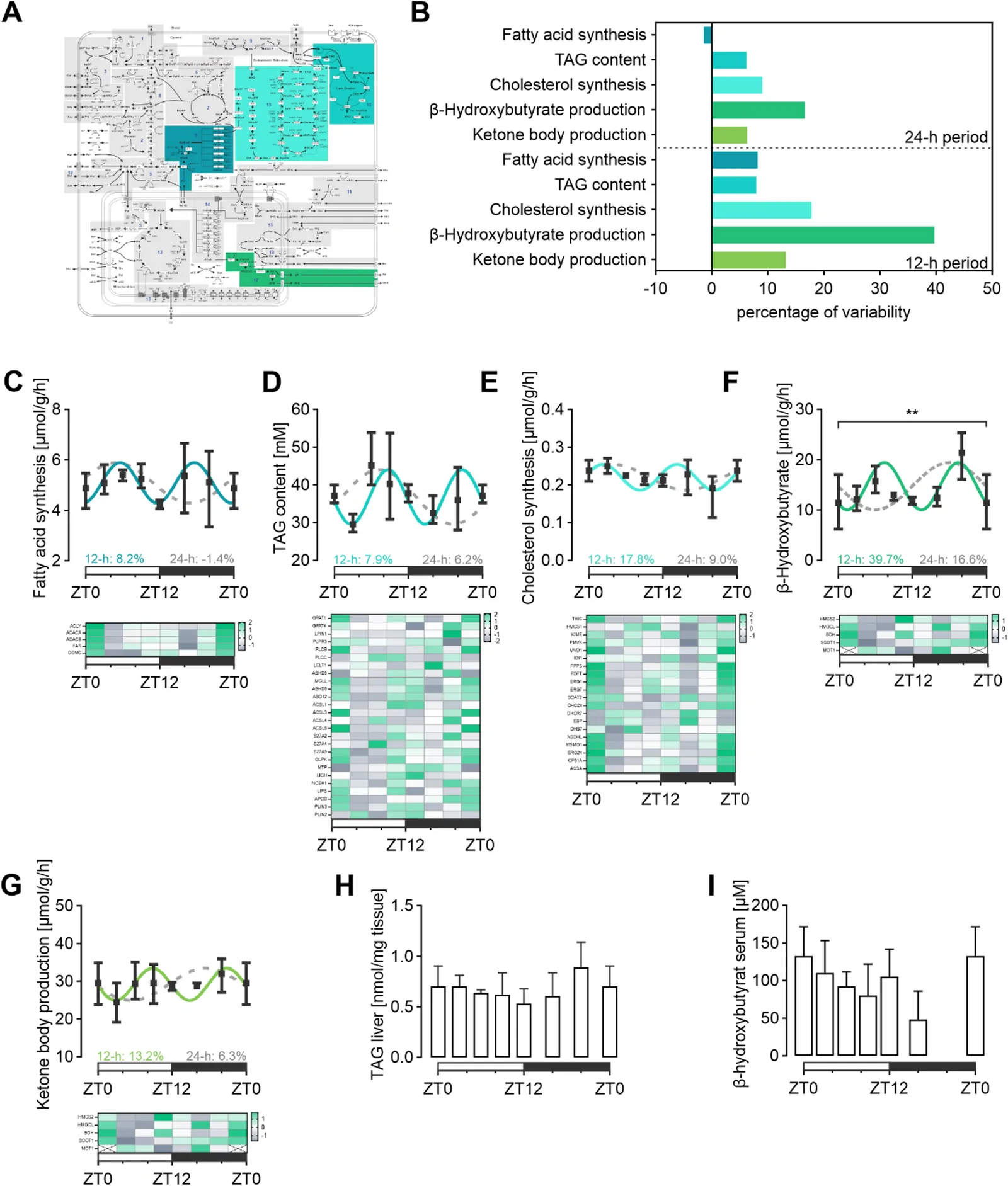
Rhythmic variations of lipid metabolism-related functions II. **A** Schematic model representation with colored pathways relevant to lipid metabolism. **B** Relative variability of metabolic functions explained by 12- or 24-hour rhythms. **C**–**G** Maximal calculated capacity of fatty acid synthesis, TAG content, cholesterol synthesis, β-hydroxybutyrate production, and ketone body production. Solid lines indicate best-fit 12-hour rhythmic functions; dashed lines indicate 24-hour fits. Below each graph, heatmaps show normalized Z-scores of protein abundances for all proteins included in the respective model module. **H** Quantified TAG content in liver tissue (*n* = 34). **I** β-hydroxybutyrate concentration in serum (*n* = 3). (C-I) Values are plotted as mean ± range. One-way ANOVA with **p < 0.01

Proteins associated with fatty acid synthesis exhibited diurnal oscillations, but capacity variability could not be explained by 12-hour or 24-hour rhythms (Fig. 4B-G). Similarly, TAG content capacity lacked clear rhythmicity, as indicated by unchanged liver TAG content (Fig. 4H). Cholesterol synthesis capacity showed diurnal oscillation, but flux was negligible, supported by unchanged serum cholesterol levels (Fig. 4E; Fig. S6 in Online Resource 1). Ketone body and β-hydroxybutyrate (BHB) production were also investigated; BHB production exhibited significant diurnal rhythmicity, explaining 40% of its variability. Although overall ketone body production showed low rhythmicity, the diurnal rhythm in BHB production likely indicates changes in mitochondrial redox state (Fig. 4B, F + G; Fig. S8D-E in Online Resource 1). However, since we found no significant correlation between ketogenic capacities and measured plasma ketone body levels or even among plasma ketone body levels, we conclude that the hepatic ketogenic capacity is not utilized under ad libitum conditions.

In summary, nearly all lipid metabolism functions, except fatty acid uptake and TAG content, exhibited synchronized rhythms, suggesting coordinated interplay among these pathways, allowing the liver to respond to changing metabolic demands throughout the day.

### Mitochondrial energy production

**Fig. 5:**
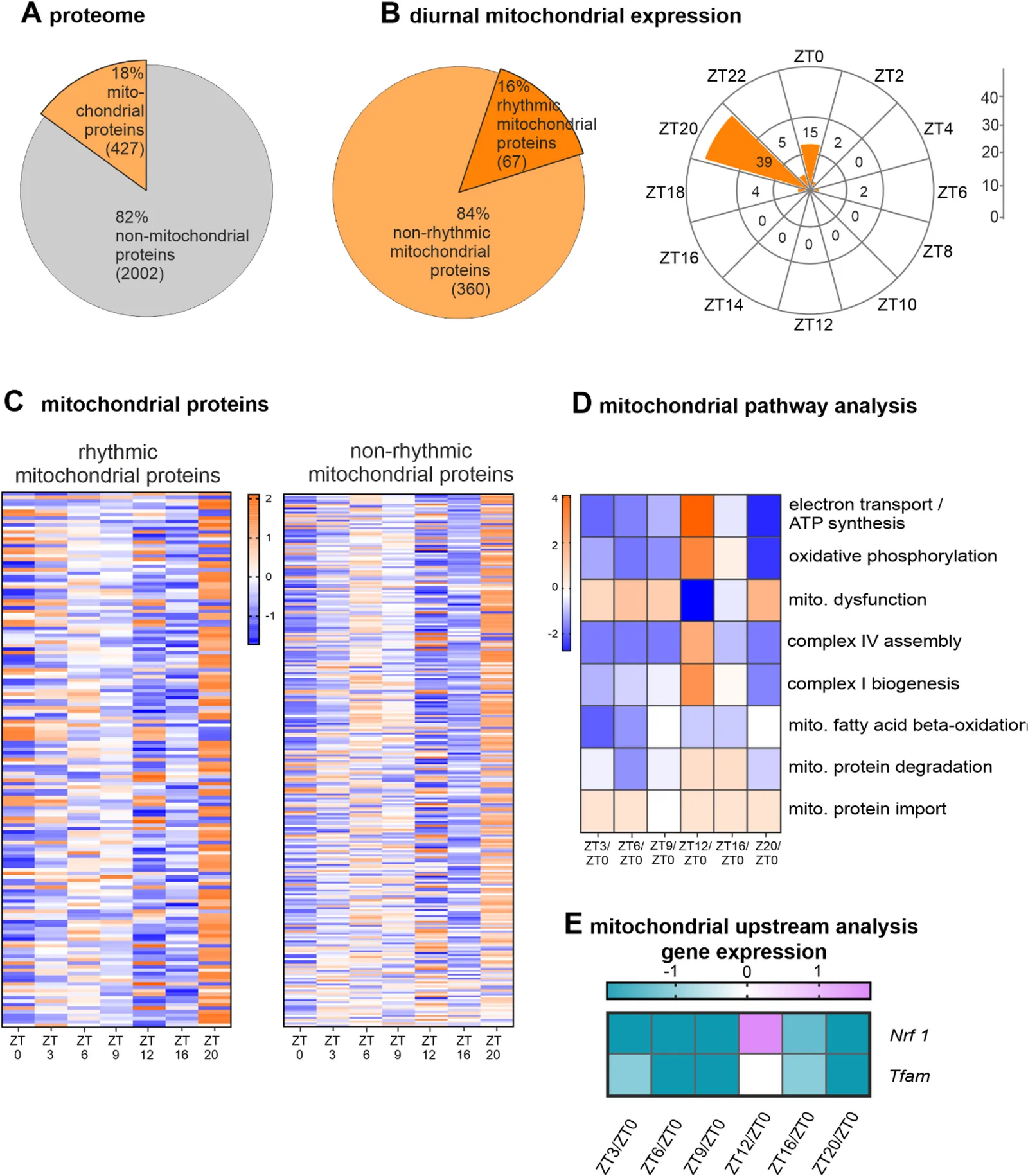
Rhythmic analysis of mitochondrial proteins and Ingenuity Pathway Analysis (IPA). **A** Distribution of the proteome, showing 18% mitochondrial proteins (427 proteins) and 82% non-mitochondrial proteins (2,002 proteins). **B** Diurnal mitochondrial protein expression. The pie chart shows that 16% of mitochondrial proteins (67 proteins) exhibit rhythmic expression, while 84% (360 proteins) are non-rhythmic. The radar plot represents the number of rhythmic mitochondrial proteins peaking at each ZT (values indicated in the plot). **C** Heatmap of z-normalized protein expression across a 24-hour cycle for rhythmic (left) and non-hythmic (right) mitochondrial proteins. Each row represents a protein, with expression levels indicated by color intensity (blue to orange), ranging from low to high expression. **D** Mitochondrial IPA based on proteomic data of mitochondrial proteins. The heatmap displays a Z-score analysis, indicating the relative activity levels of various mitochondrial pathways (e.g., electron transport/ATP synthesis, oxidative phosphorylation, fatty acid β-oxidation) across different time points (ZT0–ZT20), with each data point representing the fold change relative to ZT0. **E** Upstream regulator IPA of NRF1 and TFAM. The heatmap displays predicted activation states of transcriptional regulators based on the expression patterns of their downstream targets. Regulators with positive activation Z-scores are shown in magenta (predicted activation), while negative Z-scores are shown in green (predicted inhibition). White indicates regulators without a significant prediction

We demonstrated above that various metabolic functions exhibited distinct time-dependent rhythms, peaking at specific intervals within the diurnal cycle. A central aspect of metabolism is the supply of products that can be used for energy production in the form of ATP, which is the most important molecule needed for cell function. Therefore, we extracted mitochondrial protein data and analyzed them in more detail to better understand rhythmicity at the mitochondrial level. MitoCarta 3.0 was used to identify the mitochondrial proteins in the whole proteomic dataset [22]. BioDare2 was then used to identify significantly rhythmic proteins. Among the 2,429 proteins identified, 18% (427 proteins) were mitochondrial, and the remaining 82% (2002 proteins) were non-mitochondrial (Fig. 5A; Table S11 in Online Resource 9). We subsequently analyzed the rhythmicity of the mitochondrial proteins. Among the mitochondrial proteins, 16% (67 proteins) exhibited a significant rhythmicity, whereas 84% (360 proteins) were nonrhythmic (Fig. 5B; Table S6 in Online Resource 7). Furthermore, the levels of the most significantly rhythmic proteins peaked at ZT20 (39 proteins) and ZT0 (15 proteins).

Ingenuity Pathway Analysis (IPA) revealed the diurnal regulation of key mitochondrial pathways. Compared with ZT0, pathways such as electron transport/ATP synthesis and OXPHOS were significantly upregulated around ZT12, indicating that mitochondrial energy production was highest during this period. Conversely, mitochondrial dysfunction pathways were upregulated at ZT4 (Fig. 5D; Table S12 in Online Resource 1). To gain insight into the underlying mechanism, we performed an upstream IPA using proteomic data of mitochondrial proteins. Upstream analysis in IPA identified the transcription factors NRF1 (nuclear respiratory factor 1) and TFAM (mitochondrial transcription factor A) as significantly activated regulators, potentially contributing to the rhythmic expression of mitochondrial proteins. NRF1 showed a highly significant overlap with its known targets among the differentially expressed proteins (p-value of overlap = 1.21 × 10⁻⁹), while TFAM also displayed a significant overlap (p-value of overlap = 3.08 × 10⁻⁵). Both regulators were predicted to be activated based on positive activation z-scores, supporting their potential roles in the coordination of mitochondrial rhythmicity of functions like complex I biogenesis and ATP synthesis (Fig. 5E). NRF1 is a key regulator of mitochondrial biogenesis and respiratory gene expression, while TFAM is essential for mitochondrial DNA transcription and maintenance, suggesting that temporal control of mitochondrial function may be transcriptionally coordinated.

**Fig. 6:**
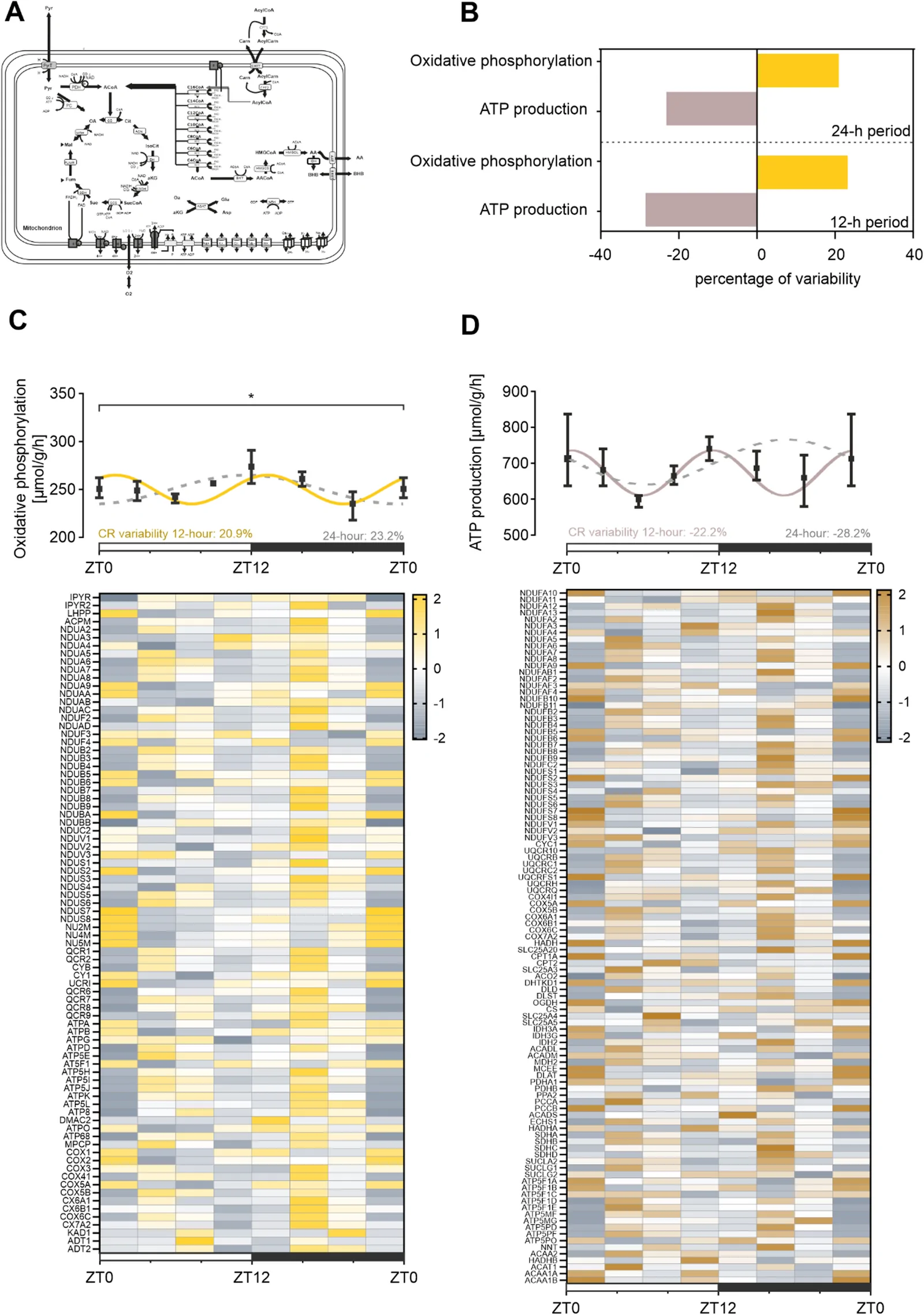
Rhythmic variations of oxygen utilization and ATP production in mitochondrial detoxification. **A** Reactions and transport processes included in the liver mitochondria kinetic model. The model comprises the following subsystems: Uptake of pyruvate and fatty acids from the cytosol in the mitochondrion, the production of acetyl-CoA from pyruvate via pyruvate-dehydrogenase (PDH) and from acyl-CoA by β-oxidation, production of NADH and FADH2 in the citric acid cycle from acetyl-CoA; the respiratory chain composed of four complexes, of which complexes I, III, and IV function as proton pumps. The resulting proton gradient and mitochondrial membrane potential (Vmm); the rate of proton-assisted ion transport of Na^+^, K^+^, Ca^2+^, and phosphate (P) across the inner mitochondrial membrane, the rate of ATP generation by the of the FOF1-ATPase, the adenine nucleotide exchanger (NE) exchanging mitochondrial ATP against cytosolic ADP; generation of ketone bodies and ketone body exchange between the mitochondrion and the cytosol. **B** Relative variability of metabolic functions explained by 12- or 24-hour rhythms. **C** + **D** Maximal calculated capacity of oxidative phosphorylation and ATP production. The solid lines depict best-fit sinusoidal wave functions with a 12-hour period, the dashed lines with a 24-hour period. Below each panel, z-score heatmaps show the normalized protein expression profiles of all model-included proteins contributing to the respective pathway. Values are plotted as mean ± range. One-way ANOVA with *p < 0.05 and **p < 0.01

The kinetic mitochondria model described in the methods section was used to examine the total metabolic capacity of mitochondria, as indicated by the rate of OXPHOS and ATP production (Fig. 6A). Surprisingly, the variability observed in both mitochondrial processes – OXPHOS and ATP production – could not be adequately explained by either a 12-hour or 24-hour rhythm. Notably, the calculated variability in ATP production exhibited a negative range, indicating that ATP production may follow any rhythm but cannot be explained by a 12-hour or 24-hour oscillation (Fig. 6B). These findings were supported by the results of the mathematical kinetic mitochondria model, which demonstrated slight diurnal variation in OXPHOS, but the variance in the individual data points was considerable, particularly for ATP production (Fig. 6C, D). However, the mathematical model revealed that ATP production was highest at ZT0 and ZT12, which corresponds with the periods when mitochondrial proteins and key energy-producing pathways were most active (Fig. S9A-F in Online Resource 1). The lowest levels of ATP production occurred around ZT6 and ZT20, highlighting a rhythmic fluctuation in mitochondrial energy output capacity (Fig. 6D). We also compared ATP production capacities between the light (ZT0–12) and dark (ZT12–0) phases but found no significant differences (Fig. S10 in Online Resource 1). This finding was not dependent on the assignment of the boundary time points ZT0 and ZT12 to either phase.

### Detoxification

**Fig. 7:**
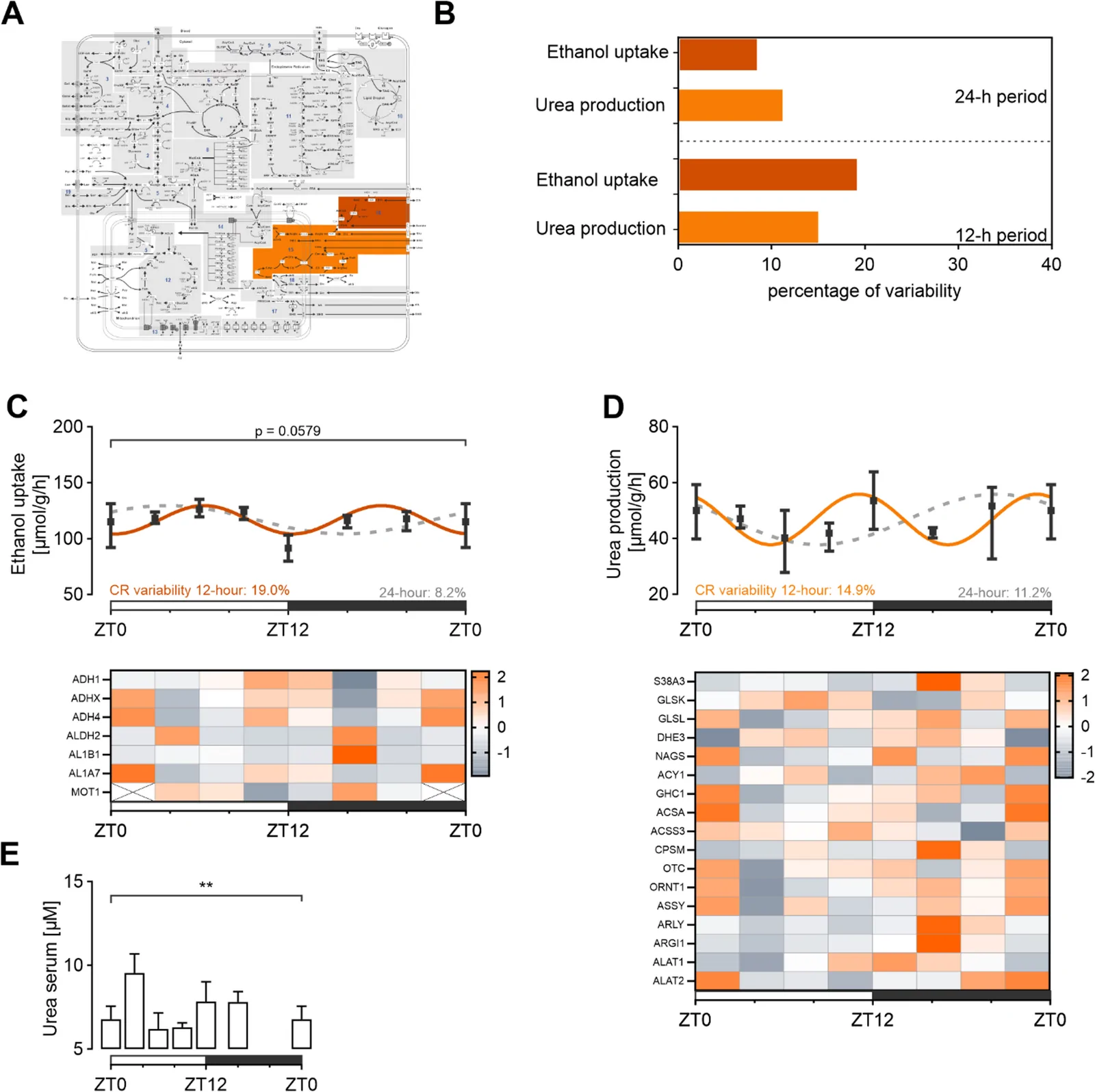
Rhythmic variations of detoxification. **A** Schematic model representation with colored pathways relevant to detoxification. **B** Relative variability of metabolic functions explained by 12- or 24-hour rhythms. **C** + **D** Maximal calculated capacity of urea production and ethanol uptake, the latter referring to the hepatic capacity for ethanol detoxification in case of alcohol exposure. The solid lines depict best-fit sinusoidal wave functions with a 12-hour period, the dashed lines with a 24-hour period. Below each panel, heatmaps display the z-score normalized protein expression of model-included proteins contributing to the respective metabolic function. **E** Urea content in serum (*n* = 3). (C-E) Values are plotted as mean ± range. One-way ANOVA with *p < 0.05 and **p < 0.01

The liver also plays a crucial role in clearing potentially harmful substances, such as ammonia and ethanol, from the body. For ethanol metabolism, 19% of the overall variability could be attributed to diurnal changes (Fig. 7B-D; Fig. S11B in Online Resource 1). Rhythmic changes over a 12-hour period contributed 15% of the variability in the maximal capacity for urea production, a key detoxification process. This capacity peaked at the light‒dark transition and reached a minimum at night (Fig. 7B + D). The rate-limiting enzyme of the urea cycle, carbamoyl-phosphate synthase (CPSM), exhibited the same rhythm (Fig. S11A in Online Resource 1). Interestingly, despite the energy requirement for ammonia detoxification, the calculated capacity for urea production was not synchronized with other energy-providing functions, such as β-oxidation. Additionally, the serum concentrations of the amino acids aspartate (*p* = 0.01431) and glutamate (*p* = 0.01764) were significantly correlated with urea production throughout the day (Fig. 7D; Table S13 in Online Resource 1). Significant fluctuations in the measured urea concentration in the serum of the mice can also be seen at different times of day, although these appear slightly offset compared to the model (Fig. 7E).

## Discussion

Metabolic processes in the liver are controlled by a complex interplay of sympathetic and parasympathetic signals synchronized with the central clock, the intrinsic circadian rhythm of the liver, and hormonal regulation. This regulation is associated with nutrient availability and behavioral patterns such as physical activity and feeding habits.

The traditional approach of evaluating circadian rhythms via changes in proteins, lipids, and metabolites from omics analyses has limited ability to explain functional effects [24–26] because measurable metabolic variables exhibit circadian fluctuations that primarily reflect nutrient availability and activity [27]. To overcome this limitation, we combined metabolomic and lipidomic analyses with mathematical modeling of liver metabolism, revealing changes in metabolic capacity due to protein levels and allowing direct assessment of the effects of circadian rhythms.

Mathematical modeling offers valuable mechanistic insights by integrating enzyme kinetics with systemic metabolic regulation, enabling functional insights and the identification of causal relationships that go beyond correlative patterns observed in omics data. This approach has been successfully applied to both preclinical models and clinical datasets, spanning physiological liver function and various pathologies including viral hepatitis, MASH, and HCC [18, 28–31]. Recently, we employed this modeling framework to simulate the effects of pharmacological interventions in the context of fatty liver disease [32].

The mice in this study had ad libitum access to food, permitting eating according to their natural circadian rhythm without artificial feeding cycles, thereby exhibiting a nocturnal bias, consistent with known feeding patterns in rodents [33, 34]. Feeding patterns are a critical determinant of hepatic metabolic rhythms, as demonstrated by Hatori et al., showing that time-restricted feeding (TRF) without caloric restriction can strongly modify metabolic cycles in mice, even under high-fat diet conditions [33]. Similarly, Vollmers et al. highlight the strong contribution of feeding patterns to the rhythmic regulation of hepatic gene expression even in clock-deficient mice [34].

Our investigation captured changes in metabolic capacity due to varying protein amounts. Importantly, plasma metabolite concentrations were not included as input for the modeling. While it is clear that variability in substrate availability strongly influences metabolic fluxes, our goal was to distinguish changes in metabolic capacities driven by proteomic alterations from the well-established effects of substrate availability on metabolic fluxes.

Nevertheless, we compared modeled capacities with measured metabolite and lipid levels and observed significant time-dependent fluctuations in plasma levels of glucose, pyruvate, fatty acids, and hepatic glycogen, in correlation with the respective metabolic capacities. Peaks in β-oxidation and VLDL export aligned with elevations in serum fatty acids, such as palmitic and linoleic acid, while changes in the capacity for glucose handling aligned with plasma pyruvate and glucose levels.

The observed correlation between capacity changes and substrate availability may reflect several underlying mechanisms. First, changes in metabolic capacities could partially translate into changes in fluxes (e.g., VLDL export), thereby supporting metabolic function in accordance with physiological demands. Given that the half-life of many metabolic enzymes ranges from 12 to 48 h, it is plausible that recurring patterns in substrate availability induce proteomic adaptations, aligning enzymatic capacities with the timing of subsequent cycles. Alternatively, these adaptations may be driven by intrinsic clock mechanisms – our current data cannot distinguish between these possibilities. Conversely, correlations with changes in plasma nutrient levels might simply indicate the liver’s relative contribution to systemic metabolite levels, rather than a coordinated systemic requirement. A more comprehensive analysis that includes other major metabolic organs – such as adipose tissue, muscle, heart, and brain – would be necessary to resolve these distinctions.

Our analysis of the rhythmicity of the entire proteome is consistent with previous findings. For example, Robles et al. reported approximately 6% of liver proteins exhibit rhythmic expression [35], whereas our study found 9.6%. Data on rhythmic metabolites (48.6%) in blood closely matched previous findings [36], with high concentrations during the light phase. The temporal distribution of peak metabolite levels was consistent with Dyar et al. [37].

Similar results were seen in liver lipidomic data, where 41% of lipids exhibited significant rhythmic behavior, mostly peaking during the day. Many rhythmic lipids are components of cell membranes, such as PCs, which correlate with hepatic high-density lipoprotein and VLDL production and secretion [38, 39].

Our kinetic modeling of glucose metabolism confirmed that both gluconeogenesis and most steps of glycolysis occur in a 12-hour rhythm [40, 41]. The rhythms of glycolysis and gluconeogenesis were approximately three hours out of phase, optimizing energy use. This pattern allows glucose supply to be maximized during periods of increased activity, such as muscle activity during the dark phase. Glycolysis peaked approximately three hours later, coinciding with the expected peak in glucose supply from feeding. The ultradian rhythm of hepatic glucose processing was reflected in plasma glucose and pyruvate concentrations and intrahepatic glycogen storage. This observation was also consistent with studies that reported that glycogen content was greatest during the transition from dark to light and almost exactly 12 h later during the transition from light to dark in ad libitum-fed nocturnal mice and rats [40].

Experimental data confirming the model results for fructose and galactose uptake are currently lacking. Evidence suggests fructose uptake oscillation is related to rhythmicity in lipid metabolism [42], but no detailed in vivo studies have been performed. The same holds for galactose uptake [43].

Lipid metabolism modeling revealed synchronous rhythms of fatty acid uptake, TAG synthesis, VLDL export, and BHB production. The ultradian rhythm was lowest during light-dark-light transitions at ZT0 and ZT12, coinciding with increased metabolic capacity as activity levels changed. Our assessment of VLDL export aligns with previous studies, indicating VLDL is directly related to plasma TAG concentration [44].

VLDL production was linked to nutrient uptake; during fasting, VLDL production increased as fatty acids were processed into TAGs, then released as VLDL. After feeding, VLDL secretion decreased. These findings were confirmed in mice subjected to a feeding regime in which they had access to food only at night and fasted during the day [44], which simulated the natural rhythm and allowed the study of its regulation in detail. Our mice had free access to food, likely leading to less precise rhythmicity than in time-restricted studies, which show clear 12- or 24-hour rhythms of VLDL export.

Mathematical modeling of fatty acid utilization confirmed its association with gluconeogenesis, highlighting their interdependence [45]. Fatty acids stimulate hepatic ATP production for gluconeogenesis, induced by glucagon at low glucose levels. Serum fatty acid levels peaked around ZT6, followed by an increase in gluconeogenic capacity after a 3-hour delay to ensure glucose supply during lower food consumption periods. The transition from gluconeogenesis to glycolysis was smooth and physiologically important, with synchronized regulatory mechanisms.

The fatty acid synthesis pathway exhibited limited diurnal regulation, primarily in response to nutrient and hormonal cues rather than the body’s internal clock. The fatty acid synthesis pathway is sensitive to sterol regulatory element-binding protein 1c (SREBP-1c), which is activated mainly by insulin and feeding [46], although some regulation by circadian signals has also been reported [47]. Additionally, fatty acid synthesis is highly sensitive to insulin through the phosphorylation of acetyl-CoA carboxylase [48], and the lack of proteomic variation when nutrients are not available, as in Berndt et al. [13], explains the observed diurnal variation [49], indicating regulation by the metabolic state over circadian rhythms. Similarly, ketone body production exhibits weak circadian regulation, as ketogenesis is linked mainly to prolonged fasting, during which fatty acids are oxidized to produce ketones as alternative energy sources [50]. In our study, however, we did not assess actual ketogenesis in vivo, but our results characterize how the liver’s ketogenic capacity fluctuates under basal conditions. Because the mice were fed ad libitum, active ketogenesis is expected to be minimal, and serum BHB levels remain largely flat – consistent with previous findings (e.g [7, 51]). Nonetheless, we observed a significant 12-hour rhythmicity in this capacity (~ 40% explained variance), likely reflecting temporal regulation of the underlying protein expression, as key enzymes of ketogenesis (D-beta-hydroxybutyrate dehydrogenase, mitochondrial; 3-hydroxy-3-methylglutaryl-CoA lyase; and 3-hydroxy-3-methylglutaryl-CoA synthase 2) are significantly associated with the ketogenic capacity (see Figs. S12-S16 in Online Resource 1). We emphasize, however, that under true fasting conditions, we would expect a substantially stronger and more sustained increase in ketogenesis over time – not a periodic variation – as hormonal and substrate signals would drive continuous induction of this pathway.

The combination of experimental proteomics and mathematical modeling in this study uncovered rhythmic patterns in mitochondrial activity, but the overall ATP production capacity displayed only weak rhythmicity and could not be statistically distinguished from random, time-independent fluctuations around a stable baseline. This is contrary to the clear rhythmicity observed in many other metabolic functions, suggesting that ATP production is a core function preserved for stability rather than subjected to anticipatory fluctuations that might compromise the liver’s energetic reserve.

Upstream analyses further identified upregulation of transcription factors such as NRF1 and TFAM – well-established regulators of mitochondrial biogenesis [52] – at the same time points as the observed mitochondrial activity. Although these factors have been associated with oscillatory behavior [53], the rhythmic patterns did not conform cleanly to canonical 12- or 24-hour cycles. Instead, the data indicate that mitochondrial dynamics in the liver might be governed by nutrient availability rather than intrinsic diurnal or circadian rhythms, consistent with previous research showing that mitochondrial behavior adapts to the metabolic state of the cell [54, 55].

This metabolic flexibility is supported by the finding that only around a third of mitochondrial proteins exhibited rhythmic expression, with the remaining two-thirds remaining constant. This reinforces the idea that functional stability is prioritized over efficiency-driven fluctuations. Mitochondrial morphology also adapts to the nutritional state: elongation occurs during periods of nutrient scarcity to maximize ATP production efficiency, whereas fragmentation is favored when nutrients are abundant to accommodate elevated metabolic demand [56, 57]. These structural shifts can occur both acutely and chronically, highlighting the dynamic, resource-responsive nature of mitochondrial function in hepatic tissue.

With respect to the metabolism of potentially harmful substances, we found that the physiological process of urea production exhibited slight diurnal behavior. Surprisingly, the expression of the rate-limiting enzyme CPSM at the protein level did not significantly change, contradicting studies demonstrating a diurnal rhythm under ad libitum feeding conditions [58, 59]. Notably, although ethanol is not a standard metabolite, its capacity in the liver exhibits a diurnal profile, suggesting varying efficiencies in ethanol metabolism throughout the day. This result is consistent with previous research indicating a day-time-dependent rhythm in the expression of ethanol-metabolizing genes, such as alcohol dehydrogenase and aldehyde dehydrogenase [60]. The rhythms of xenobiotic metabolism are particularly crucial in the metabolism of drugs and can dramatically influence their efficacy, making further knowledge of these rhythms essential for effective humane therapies [61].

Compared to the conventional 24-hour cycle, our analyses suggest that the liver exhibits a greater likelihood of 12-hour rhythmicity in metabolic processes, as previously suggested [62]. Our findings also align with those of Cretenet et al., who found 12-hour rhythmicity in lipid metabolism [63]. Additionally, our data support effective metabolic coordination, particularly in glucose metabolism. Our findings align with the vehicle-cargo hypothesis of Pan et al., emphasizing distinct functions in 12-hour and 24-hour periods [64]. Importantly, while rhythmic variations in metabolic capacity contribute to fine-tuning and have the potential to optimize metabolic activity, these variations are not the sole or even the most significant regulators. Factors such as diet, physical activity, hormonal status, and regulation by the central nervous system potentially play more pivotal roles, as they enable rapid responses to external triggers. Nonetheless, functions such as glucose handling, fatty acid uptake, TAG synthesis, β-oxidation, and energy metabolism exhibit robust 12-hour rhythms, accounting for 25–40% of total variability.

Mechanistically, recent evidence indicates that the hepatic 12-hour transcriptional program is not merely a sub-harmonic of the canonical 24-hour BMAL1/CLOCK circadian loop but constitutes a genetically and functionally distinct oscillatory system. This 12-hour rhythm is driven primarily by metabolic and stress-related cues – particularly twice-daily surges in nutrient flux and ER stress associated with feeding–fasting transitions. The ER stress response factor XBP1s (X-box binding protein 1, spliced isoform) has emerged as a central regulator: both XBP1s and hundreds of its lipid-handling target genes exhibit robust 12-hour oscillations, and hepatocyte-specific deletion of Xbp1 results in the loss of approximately 86% of all 12-hour rhythmic transcripts while leaving 24-hour circadian gene expression largely unaffected [64]. In contrast, BMAL1, a core transcription factor of the 24-hour circadian machinery, is not required for the expression of many 12-hour genes. BMAL1-deficient mice retain pronounced 12-hour rhythms in transcripts involved in mRNA processing, protein turnover, and mitochondrial dynamics – further supporting the notion that the 12-hour program is a distinct transcriptional layer rather than a harmonic output of the circadian clock [65]. Together, these findings support the existence of a genetically separable and metabolically important 12-hour oscillator in the liver that functions independently of the classical circadian network.

### Translational implications

Understanding hepatic rhythmicity is crucial, as disruptions in lipid metabolism have been linked to diseases such as MASH, even with a resilient core circadian clock [66]. The 12-hour oscillations we report appear to be largely plastic and responsive to external metabolic cues – particularly nutrient-related signals such as insulin, glucose, and circulating fatty acid levels – rather than being hard-wired into the core molecular clock machinery. This regulatory flexibility allows the 12-hour program to adapt to light–dark/eating transitions but also renders it susceptible to disruption under conditions of circadian misalignment or metabolic stress.

Indeed, human studies show that circadian misalignment, as seen in shift workers, leads to significant alterations in metabolic markers like insulin sensitivity and glucose homeostasis [67], which likely interfere with the normal ultradian rhythmicity of metabolic processes. Importantly, while the expression of core molecular clock genes remains relatively stable, time-of-day-dependent gene expression patterns in the human liver become markedly disrupted as metabolic dysfunction-associated steatotic liver disease (MASLD) progresses to MASH [66]. This link between rhythm disruption and disease progression is further supported by mechanistic studies in a humanized liver mouse model, where the combination of MASLD and chronic circadian disruption (via repeated jet lag) accelerates the development of advanced liver disease, including cirrhosis and hepatocellular carcinoma [68]. Additionally, epidemiological data consistently associate shift work with increased risk for type 2 diabetes and MASLD [69–71], reinforcing the notion that perturbations in temporal metabolic regulation – including 12-hour rhythms – may contribute directly to disease pathogenesis.

Recognizing 12-hour metabolic oscillations as a distinct regulatory layer in hepatic physiology opens up new possibilities for translational applications. If the liver organizes key metabolic functions, such as glucose handling, lipid metabolism, and mitochondrial activity, in biphasic patterns aligned with recurring light–dark/eating transitions, it becomes plausible that therapeutic interventions could be optimized by aligning with these intrinsic temporal structures.

Chronotherapy, i.e., time-of-day-dependent drug administration, has mainly considered circadian (24-hour) cycles to date. However, emerging evidence suggests that ultradian (12-hour) rhythmicity may also influence pharmacodynamics, particularly in metabolically active organs such as the liver. For example, metformin, a key drug in the treatment of type 2 diabetes, exhibits time-dependent variations in its glucose-lowering effects in rodents, which are associated with differential activation of hepatic AMP-activated protein kinase depending on the timing of administration [72]. However, chronic treatment with metformin seems not to disrupt the hepatic circadian clock in mice [73]. In humans, its pharmacokinetics vary throughout the day, partly due to circadian modulation of renal clearance and hepatic transporter expression [74]. Although a direct interaction between metformin and hepatic 12-hour rhythms has not yet been demonstrated, these findings suggest that such relationships may exist and warrant systematic investigation.

In this context, future studies should explore whether hepatic 12-hour programs modulate the temporal responsiveness to drugs such as metformin. A comprehensive assessment of time-resolved drug target expression, phase-specific efficacy under defined feeding/fasting conditions, and potential disruptions of these rhythms in individuals with altered behavioral schedules – such as shift workers or patients with metabolic disease – could provide key insights. These efforts may ultimately help to identify optimal time windows for pharmacological intervention, thereby improving treatment outcomes and minimizing side effects. Similarly, TRF protocols may benefit from alignment with natural 12-hour hepatic rhythms. Previous studies have shown that TRF enhances hepatic metabolic rhythms and improves systemic insulin sensitivity in mice, even without caloric restriction [33, 75]. Moreover, TRF has recently been proposed as a promising dietary intervention for MASLD patients [76]. Tailoring TRF or drug administration to reinforce or synchronize with ultradian hepatic programs may thus represent a novel strategy for precision chronotherapy in metabolic disorders, including type 2 diabetes, MASLD/MASH, and obesity.

### Limitations

In the present work, we restricted the cohort to male C57BL/6 N mice to avoid the additional layer of variability introduced by the 4- to 5-day estrous cycle. Fluctuating estrogen and progesterone levels modulate both the central and hepatic clock machinery and can shift the phase and amplitude of rhythmic outputs, such as food intake, locomotor activity, and liver gene expression [77, 78]. Therefore, including only males allowed for a tighter resolution of proteome-function relationships with a practical sample size. However, we acknowledge that this design limits generalizability because sex steroids themselves act as zeitgebers and shape metabolic rhythms [79]. Future studies should compare females across defined estrous stages or use ovariectomized/hormone replacement models to determine the extent to which the 12-h rhythms reported here are conserved, blunted, or reshaped in females.

Another limitation of our study is the use of a fixed 12-hour light/12-hour dark cycle without exploring alternative environmental entrainment conditions. While this standardized protocol allowed for controlled analysis of temporal changes in protein abundance, it does not fully disentangle intrinsic 12-hour rhythms from those potentially influenced by external cues such as feeding behavior or light exposure. Although our focus was on modeling time-resolved metabolic capacities based solely on protein abundance – independent of fluctuating nutrient and hormonal states – we recognize that environmental light/dark and feeding cues can influence hepatic metabolism and may themselves exhibit rhythmic patterns. This might also limit the direct transferability of our findings to humans, where typical light/dark cycles (e.g., 8/16 h) and lifestyle-driven feeding and activity patterns may shift or reshape hepatic metabolic rhythms. Understanding how intrinsic liver oscillations integrate with human-specific external cues remains an important direction for future research.

Finally, while our mathematical modeling approach offers a powerful means to infer rhythmic changes in metabolic capacities, these should not be equated with actual metabolic fluxes. Fluxes represent the realized activity of these capacities under specific physiological conditions. For instance, although the liver maintains the capacity to produce ketone bodies, this pathway remains inactive under well-fed conditions. Similarly, while the liver always possesses the enzymatic machinery for both glucose uptake and gluconeogenesis, only one of these processes dominates at a given time. Accurately determining the actual flux at any specific time point would require additional contextual information, including plasma nutrient levels, perfusion rates, hormonal states, intracellular signaling, and neural input.

### Conclusion

Our investigation using the HEPATOKIN1 model revealed that nearly all energetically relevant hepatic metabolic processes follow a rhythmic pattern, with many exhibiting a 12-hour cycle. These findings underscore the liver’s flexibility in regulating metabolic rhythms, with implications for future liver research and the development of therapeutic interventions aimed at optimizing metabolic health. The kinetic modeling used in our study provides a unique opportunity to investigate liver metabolic pathways’ circadian rhythms and their fine-tuning under real conditions, such as ad libitum feeding. Unlike previous approaches using constant darkness or TRF, kinetic modeling allows the simulation of liver metabolism in natural, unrestricted settings. This model offers insights into how liver metabolism operates holistically under physiological conditions for nutrient availability. Future applications of kinetic modeling could explore how dietary interventions, such as fasting or specialized diets, affect liver metabolism at a circadian level, aiding the development of personalized nutritional strategies to optimize natural circadian rhythms and prevent metabolic disorders. Kinetic models like HEPATOKIN1 represent valuable tools for avoiding costly experiments by enabling straightforward simulations and illustrating interorgan metabolic interdependence.

## Supplementary Information

Below is the link to the electronic supplementary material.

Supplementary Material 1 Online Resource 1 (ESM_1): Primary supplemental PDF (supplementary methods, figures, and tables).

Supplementary Material 2 Online Resource 2 (ESM_2): Table S1. Proteome data.

Supplementary Material 3 Online Resource 3 (ESM_3): Table S2. Lipidome data.

Supplementary Material 4 Online Resource 4 (ESM_4): Table S3. Metabolome data.

Supplementary Material 5 Online Resource 5 (ESM_5): Table S4. Metabolic functions (within physiological range or maximal capacity) and all significantly associated proteins with their p-values.

Supplementary Material 6 Online Resource 6 (ESM_6): Table S5. Comparative model fits for rhythmicity analysis of metabolic functions: constant vs. 12 h and 24 h sinusoidal models.

Supplementary Material 7 Online Resource 7 (ESM_7): Table S6. Biodare2 Analysis of all OMIC data.

Supplementary Material 8 Online Resource 8 (ESM_8): Table S7. Pearson’s correlation coefficients and corresponding p-values for ZT0-ZT16.

Supplementary Material 9 Online Resource 9 (ESM_9): Table S11. Mitochondrial proteins selected via MitoCarta 3.0.

## Data Availability

The data that supports the findings of this study are available in the Online Resources of this article.

## Abbreviations

ACLS1/3/5: acyl-CoA synthetase long chain family member 1/3/5
Adh: alcohol dehydrogenase
Aldh: aldehyde dehydrogenase
APOB: apolipoprotein B-100
BHB: β-hydroxybutyrate
CPSM/*Cps1*: carbamoyl-phosphate synthase
CPT1A: carnitine palmitoyltransferase 1A
ECHM/*Echs1*: enoyl-CoA hydratase
F16P1: fructose-1,6-bisphosphatase
G6PC: glucose-6-phosphatase
GLPK/*Gk*: glycerol kinase
GPAT1/*Gpam*: glycerol-3-phosphate acyltransferase 1
GTR2/*Slc2a2*: glucose transporter 2
HCC: hepatocellular carcinoma
IPA: Ingenuity Pathway Analysis
K6PL: 6-phosphofructokinase
KPYM/*Pkm*: pyruvate kinase (muscle)
KPYR/*Pklr*: pyruvate kinase (liver and blood cell)
LDHA: lactate dehydrogenase A
LICH/*Lipa*: lysosomal acid lipase/cholesteryl ester hydrolase
MASLD: metabolic dysfunction-associated steatotic liver disease
MASH: metabolic dysfunction-associated steatohepatitis
NRF1: nuclear respiratory factor 1
OXPHOS: oxidative phosphorylation
PC(s): phosphatidylcholine(s)
PCKGC/*Pck1*: phosphoenolpyruvate carboxykinase
PLIN3: perilipin-3
PYC: pyruvate carboxylase
S27A2/5: long-chain fatty acid transport protein/very long-chain acyl-CoA synthetase
TAG: triglyceride
TFAM: mitochondrial transcription factor A
THIM/*Acaa2*: 3-ketoacyl-CoA thiolase
TRF: time-restricted feeding
VLDL: very low-density lipoprotein
XBP1(s): X-box binding protein 1 (spliced isoform)
ZT: Zeitgeber Time
