# Supplementary material for: Modeling the dynamics of hepatic metabolism: the predominance of 12-hour rhythmicity in metabolic adaptation
Source: Cell Mol Life Sci. 2026 Jan 9;83(1):55. doi: 10.1007/s00018-025-06046-4 (PMC12819941; doi:10.1007/s00018-025-06046-4)
Supplement: Supplementary file 5 — Supplementary Material 5 Online Resource 5 (ESM_5): Table S4. Metabolic functions (within physiological range or maximal capacity) and all significantly associated proteins with their p-values. [file 18_2025_6046_MOESM5_ESM.pdf]

SUPPLEMENTARY MATERIAL

MODELING THE DYNAMICS OF HEPATIC METABOLISM: THE PREDOMINANCE OF 12-HOUR RHYTHMICITY IN METABOLIC ADAPTATION

Cellular and Molecular Life Sciences

Madlen Matz-Soja and Christiane Körner, Fritzi Ott, Janett Fischer, Eugenia Marbach-Breitrück, Christian Bergmann, Ute Hofmann, Andrej Shevchenko, Iwona Wallach, Kathrin Textoris-Taube, Michael Mülleder, Rolf Gebhardt, Thomas Berg, Nikolaus Berndt

*Corresponding author:
Nikolaus Berndt
German Institute of Human Nutrition Potsdam-Rehbruecke (DIfE)
Department of Molecular Toxicology
Nuthetal, Germany
ORCID: 0000-0001-5594-9940 | nikolaus.berndt@dife.de

TABLE OF CONTENTS

SUPPLEMENTARY METHODS 2
- Shotgun proteome profiling and data analysis 2
- Lipidome analysis 3
- Metabolome analysis 4
- Assessment of metabolic capacities 4
- Mitochondrial model description 6

SUPPLEMENTARY FIGURES 7
SUPPLEMENTARY TABLES 24
SUPPLEMENTARY REFERENCES 25

SUPPLEMENTARY MATERIAL

SUPPLEMENTARY METHODS

Shotgun proteome profiling and data analysis

The liver was homogenized in 8 M Urea containing 20 mM Hepes (pH 8.0) before samples were sonicated by three pulses of 10 s at an amplitude of 20% and centrifuged for 15 min at 20,000 rcf at room temperature. Proteins were first reduced by the addition of dithiothreitol to a final concentration of 5 mM and incubated for 30 min at 55°C, 700 rpm and then alkylated by addition of chloroacetamide to a concentration of 10 mM and incubation for 15 min at room temperature. The samples were diluted to a urea concentration of 4 M, and 1 mg of protein according to their protein determination with Pierce 660 nm (Art. 22660) was digested with lysyl endopeptidase (1:250, w/w; Wako) for 4 hours at 37°C. After further dilution to 2 M urea, proteins were digested with trypsin (1:200, w/w; Promega) overnight at 37°C. The resulting peptide mixture was acidified by the addition of TFA, and by centrifugation at 7000 rcf for 15 min at room temperature insoluble components were removed. Peptides were purified by using SampliQ C18 columns 100 mg (Agilent), lyophilized, and stored at −80°C for LC-MS/MS analysis. After resolving the peptides in loading solvent A [0.1% TFA in water/ACN (98:2, v/v)], the peptide concentration was determined with Pierce Quantitative Fluorometric Peptide Assay (Art. 23290) to inject 1.5 µg of peptides for LC-MS/MS analysis on an Ultimate 3000 RSLCnano system on-line connected to a Q-Exactive Plus mass spectrometer (Thermo Fisher Scientific). Digests were trapped on a guard column (PepMap C18, 5 mm × 300 µm × 5 µm, 100 Å, Thermo Fisher Scientific) and eluted through an analytical nanoLC column (75 µm inner diameter × 500 mm nano Acclaim PepMap C18, 2 µm; 100 Å; Thermo Fisher Scientific). Briefly, 1.5 µg of peptides were separated with a 310-min run, with the proportion of buffer B increasing from 3 to 28% in 230 min and decreasing to 12% over 30 min at a flow rate of 300 nL/min. Solvent A was 0.1% formic acid, and solvent B was 80% acetonitrile, 20% water, and 0.1% formic acid. Nanoelectrospray was generated by applying 3.5 kV. A cycle of one full Fourier transformation scan mass spectrum (300−1600 m/z, resolution of 70,000 at m/z 200, AGC target 3e6) was followed by 12 data-dependent MS/MS scans (resolution of 17,500, AGC target 5e4) with a normalized collision energy of 27. To avoid repeated sequencing of the same peptides, a dynamic exclusion window of 60 sec was used. In addition, only peptides with charge states between two and eight were sequenced.

The raw MS data were processed with MaxQuant software (1.6.0.1) utilizing the Andromeda search engine and the mouse UniProtKB, which contains 17,016 reviewed entries released in 03/2019. A false discovery rate of 0.01 for proteins and peptides, a minimum peptide length of seven amino acids, and a mass tolerance of 10 ppm for precursor ions and 20 ppm for fragment ions were needed. A maximum of two missed cleavages in the tryptic digest was acceptable. Cysteine carbamidomethylation was set as a fixed modification, whereas N-terminal acetylation, methionine oxidation, and asparagine and glutamine deamidation were

set as variable modifications. The complete proteome dataset can be found in Table S1 (see ESM_2).

Lipidome analysis

Liver tissue samples isolated at ZT0, ZT3, ZT6, ZT9, ZT12, and ZT16 were used for lipidome analysis. Lipids were extracted from serum via a modified version of the protocol by Folch et al. [1] and analyzed via shotgun mass spectrometry as described previously [2]. Briefly, liver tissue (an amount equivalent to 10 mg of total protein) was dissolved in 200 mL of ammonium bicarbonate solution (150 mM). For the subsequent quantification, 10 mL of an internal standard mixture was added (20 pmol TAG 12:0-12:0-12:0, 20 pmol DAG 17:0-17:0, 40 pmol diethyl PC 18:0-18:0, 50 pmol diethyl PE 20:0-20-0, 10 pmol PG 17:0-17-0, 40 pmol PS 12:0-12:0, 50 pmol PI 16:0-16-0, 40 pmol LPC 12:0, 40 pmol LPE 14:0, 30 pmol SM d18:1-12:0, 90 pmol CE 12:0, 20 pmol CER d18:1-12:0, 50 pmol cholesterol d7; Avanti Polar Lipids, Inc., Alabaster, AL, United States). Then, 265 mL of methanol and 730 mL of chloroform were added, and the mixture was vortexed for 1 h at 4°C. The lower organic phase was collected and dried in a vacuum centrifuge, and the lipid extracts were redissolved in a 120 mL chloroform:methanol [1:2 (v/v)] mixture. The analysis was performed in both negative and positive ion modes. For negative mode analyses, 10 mL of extract was mixed with either 12 mL of 13 mM ammonium acetate in isopropanol or 0.1% (v/v) triethylamine in methanol. For positive mode analyses, 10 mL of extract was mixed with 90 mL of 6.5 mM ammonium acetate in isopropanol before infusion. The analyses were performed on a Q-Exactive mass spectrometer (Thermo Fisher Scientific, Germany) equipped with a TriVersa NanoMate robotic nanoflow ion source (Advion BioSciences, Ithaca, NY, United States). High-resolution (140,000 at m/z 200) FT–MS spectra were acquired for 1 min within the ranges of m/z 420–1000 in negative mode and m/z 450–1000 in positive mode. Cholesterol (CHOL) was quantified as previously described [3]. Briefly, 30 mL of extract was dried under vacuum, 75 mL of acetyl chloride:chloroform [1:2 (v/v)] was added, and the mixture was incubated for 1 h at room temperature. The mixture was dried under vacuum, and the extracts were redissolved in 60 mL of chloroform:methanol [1:2 (v/v)]. A total of 10 mL of extract was mixed with 90 mL of 6.5 mM ammonium acetate in propanol before infusion and analyzed in positive ion mode. The following lipid classes were identified and quantified using LipidXplorer software [4]: triglycerides (TAGs), diglycerides (DAGs), CHOL, sphingomyelins (SMs), phosphatidylcholines (PCs), phosphatidylethanolamines (PEs), phosphatidylinositol (PI), lysophosphatidylcholine (LPC), lysophosphatidylethanolamine (LPE), and ceramides (CERs). The concentrations were recorded as the average of the biological replicates (n = 2 technical and N = 3 biological replicates), and all the data are presented in Table S2 (see ESM_3).

SUPPLEMENTARY MATERIAL

Metabolome analysis

Serum samples at ZT0, ZT3, ZT6, ZT9, ZT12, and ZT16 were used for metabolome analysis. The levels of proteinogenic amino acids, urea, pyruvate, hydroxybutyrate, fumarate, ketoglutarate, malate, and citrate were determined via GC–MS analysis as described elsewhere [5, 6]. The levels of bile acids were determined by negative electrospray ionization (ESI) LC–MS/MS in multiple reaction monitoring (MRM) mode on an Agilent 6460 triple quadrupole mass spectrometer (Agilent, Waldbronn, Germany) coupled to an Agilent 1200 HPLC system. The bile acids tauromuricholate and taurocholate were separated on a Poroshell 120 EC-C18 column (100 × 2.1 mm, 2.7 µm particle size, Agilent) with a gradient of mobile phases A (12 mm ammonium acetate in water) and B (acetonitrile) at a flow rate of 0.5 ml/min. The MRM transitions were 514.3/80 for taurocholate and tauromuricholate and 518.3/80 for the internal standards [2H4]tauromuricholate and [2H4]taurocholate. The statistical and enrichment analyses of the results were performed with MetaboAnalyst R software. After normalization, the data were log-transformed. The metabolic pathway-associated metabolic library was utilized for the quantitative enrichment analysis. A global test was used to analyze the Q statistic for each metabolite set (Table S3, see ESM_4).

Assessment of metabolic capacities

We used HEPATOKIN1 [7] in combination with a detailed model of lipid droplet metabolism [7], as described by Berndt et al. [8] to evaluate the functional implications of diurnal changes in protein abundance in the liver. The model comprises the central hepatic metabolic pathways of glycolysis; gluconeogenesis; glycogen synthesis; glycogenolysis; fructose metabolism; galactose metabolism; the creatine phosphate/ATP shuttle system; the pentose phosphate cycle, including the oxidative and non-oxidative branches; the citric acid cycle; the malate aspartate redox shuttle; the glycerol-3-phosphate shuttle; the mitochondrial respiratory chain; beta-oxidation of fatty acids; fatty acid synthesis; ketone body synthesis; CHOL synthesis; TAG synthesis and degradation; TAG synthesis and hydrolysis; very low-density lipoprotein (VLDL) synthesis and export; the urea cycle; metabolism of the amino acids serine, alanine, glutamate, glutamine, and aspartate; and ethanol detoxification (Supplementary Fig. S1).

The model includes the key electrophysiological processes of the inner mitochondrial membrane, including the mitochondrial membrane potential, mitochondrial ion homeostasis, and generation and utilization of the proton motive force as described by the Goldman–Hodgkin–Katz kinetic equations [9]. The metabolic model is coupled to a phenomenological model including hormonal signaling by glucagon and the effect of insulin on the short-term regulation of metabolic enzymes by reversible phosphorylation. Plasma glucose, fatty acid, insulin, and glucagon concentrations are not independent but are tightly linked through the pancreatic and adipose tissue-controlled release of hormones and fatty acids; therefore, we used phenomenological transfer functions describing the dependence of the plasma

concentrations of insulin, glucagon and free fatty acids on plasma glucose levels. The plasma concentrations of insulin and glucagon were directly translated into the phosphorylated state of interconvertible enzymes via a phenomenological function described by Bulik et al. [10]. Individual instantiations of the model for each sample were obtained with the protein intensity profiles determined via quantitative shotgun proteomics to scale the maximal activities of enzymes and transporters according to the relationship $v_{max}^{sample} = v_{max}^{mean\ control} \frac{E^{sample}}{E^{mean\ control}}$. The maximal activities $v_{mx}^{mean\ control}$ for the control tissue were obtained from previous studies [10]. External plasma glucose concentrations ranging from 3 mM (corresponding to a severely fasted state with high glucagon, low insulin, and high fatty acid levels) to 12 mM (corresponding to a well-fed state with low glucagon, high insulin, and low fatty acid levels) enabled the evaluation of hepatic metabolic functions in the full physiological range between low glucose and high glucose. The model describes the uptake, metabolism, and generation of glucose, fructose, galactose, pyruvate, lactate, glycerol, ammonia, serine, alanine, glutamate, glutamine, fatty acids, ethanol, acetate, urea, acetoacetate, β-hydroxybutyrate (BHB), oxygen, and VLDL particles [7]. For a detailed description, see Berndt et al. (2018, 2017) [7, 11]. Maximal capacities for fructose, galactose, and ethanol detoxification were assessed by individually varying the concentrations of the respective metabolites in the plasma. For details, see Berndt et al. [7].

Diurnal rhythm for a metabolic function were defined by minimizing square distance between a sinusoidal function with a period with an integer divisor of 24, an offset equal to the mean of the metabolic function and an amplitude equal to the standard deviation of the metabolic function, to the values of the metabolic function for all samples. Optimal constant value for a metabolic function were defined by minimizing the square distance between a constant function and the values of the metabolic function for all samples. The CR variability describes the part of the square distance of the constant function that can be attributed to the circadian rhythm, i.e. the difference between variability of the data with respect to the optimal circadian rhythm and the variability with respect to the optimal constant function. A negative value indicates that random variation around a constant metabolic function explains more variability than a circadian rhythm. Significantly regulated functional proteins were determined by a linear regression model assessing the association between maximal metabolic capacities or circadian metabolic capacities and the abundance of proteins belonging to the respective pathway (Table S4, see ESM_5). Group values were checked for normality by one-sample Kolmogorov-Smirnov test. Significant differences between groups were assessed by two-sided t-test for normal distributed group values, otherwise, Wilcoxon signed-ranked test was used. All model simulations were performed using MATLAB, Release R2023a, The MathWorks, Inc., Natick, Massachusetts, United States.

SUPPLEMENTARY MATERIAL

Mitochondrial model description

The kinetic model comprises the major metabolic pathways of mitochondrial energy metabolism from pyruvate and fatty acids (Fig. 6A). The model also contains key electrophysiological processes of the inner mitochondrial membrane, including the membrane transport of various ions, the mitochondrial membrane potential and the generation and utilization of the proton motive force. The time-dependent variations in model variables (= concentrations of metabolites and ions) are governed by first-order differential equations. The time variations of small ions were modeled via Goldman–Hodgkin–Katz kinetic equations [9]. The kinetic rate equations and maximal enzyme activities (Vmax values) for the individual samples are identical to the respective processes described in HEPATOKIN1 [7]. Energetic capacities were evaluated by computing the changes in the metabolic state elicited by an increase in the ATP consumption rate above the resting value. The ATP consumption rate was modeled by the generic hyperbolic rate law $v_{ATP}=k_{load}\cdot ATP/(ATP+K_m)$. The parameter k_{load} was increased stepwise until the ATP production rate converged to its maximal value [12].

SUPPLEMENTARY MATERIAL

SUPPLEMENTARY FIGURES

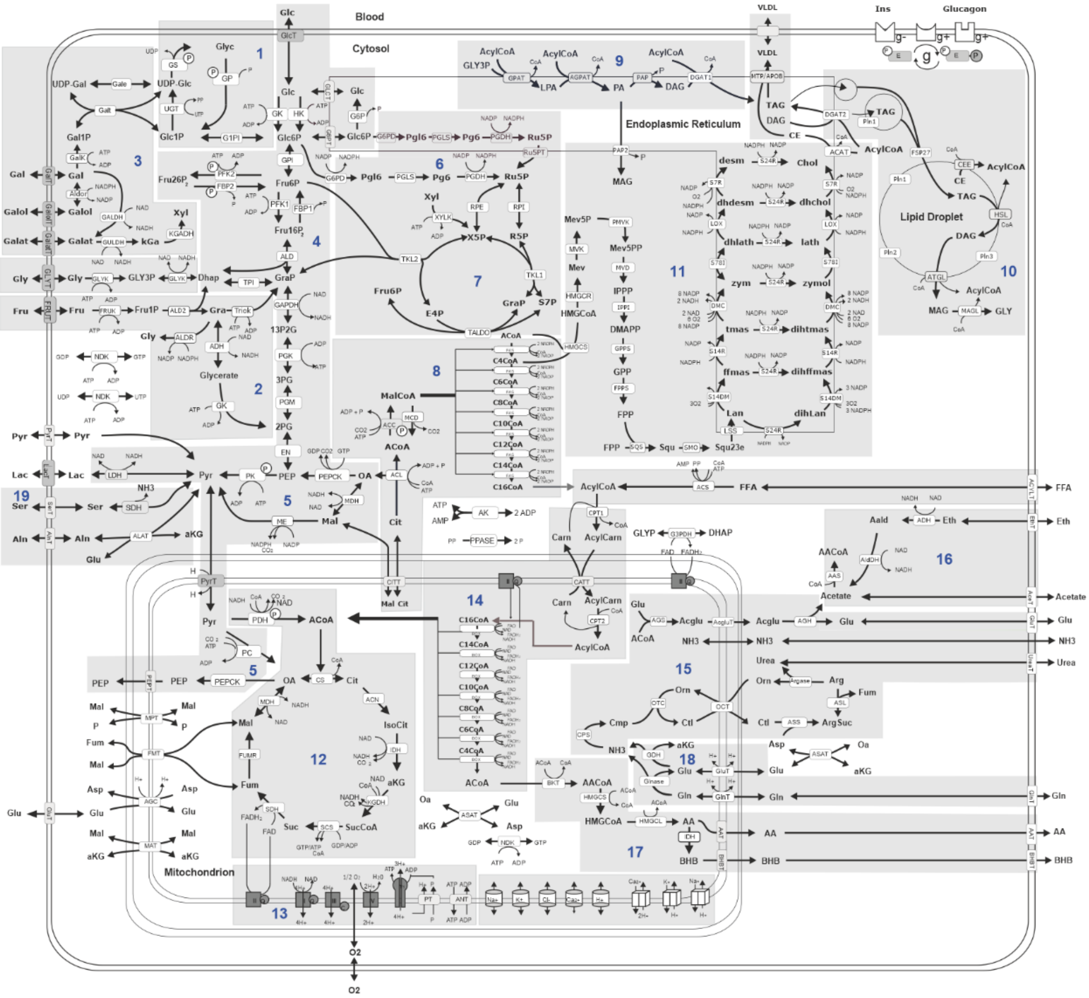

Fig. S1. Schematic model representation. Reactions and transport processes between compartments are symbolized by arrows. Single pathways as defined in biochemical textbooks: (1) glycogen metabolism, (2) fructose metabolism, (3) galactose metabolism, (4) glycolysis, (5) gluconeogenesis, (6) oxidative pentose phosphate pathway, (7) non-oxidative pentose phosphate pathway, (8) fatty acid synthesis, (9) TAG synthesis, (10) synthesis and degradation of lipid droplets and synthesis of VLDL lipoprotein, (11) cholesterol synthesis, (12) tricarbonic acid cycle, (13) respiratory chain and oxidative phosphorylation, (14) β-oxidation of fatty acids, (15) urea cycle, (16) ethanol metabolism, (17) ketone body synthesis, (18) glutamine synthesis and (19) serine and alanine utilization. Lipid droplet synthesis and degradation pathways include *de novo* synthesis of lipid droplets, lipid droplet filling, lipid droplet growth and fusion as well as lipid droplet degradation in dependence on regulatory surface proteins. Small cylinders and cubes symbolize ion channels and ion transporters. Double arrows indicate reversible reactions, which may proceed in both directions according to the value of the thermodynamic equilibrium constant and cellular concentrations of their reactants. Reactions are labeled by the short names of the catalyzing enzyme or membrane transporter given in the small boxes attached to the reactions arrow. Metabolites are denoted by their short names. The figure was adapted from Berndt et al. (2018, 2022) [7, 8]. Full names of metabolites and kinetic rate laws of reaction rates are outlined by Berndt et al. [7] and Wallstab et al. [11].

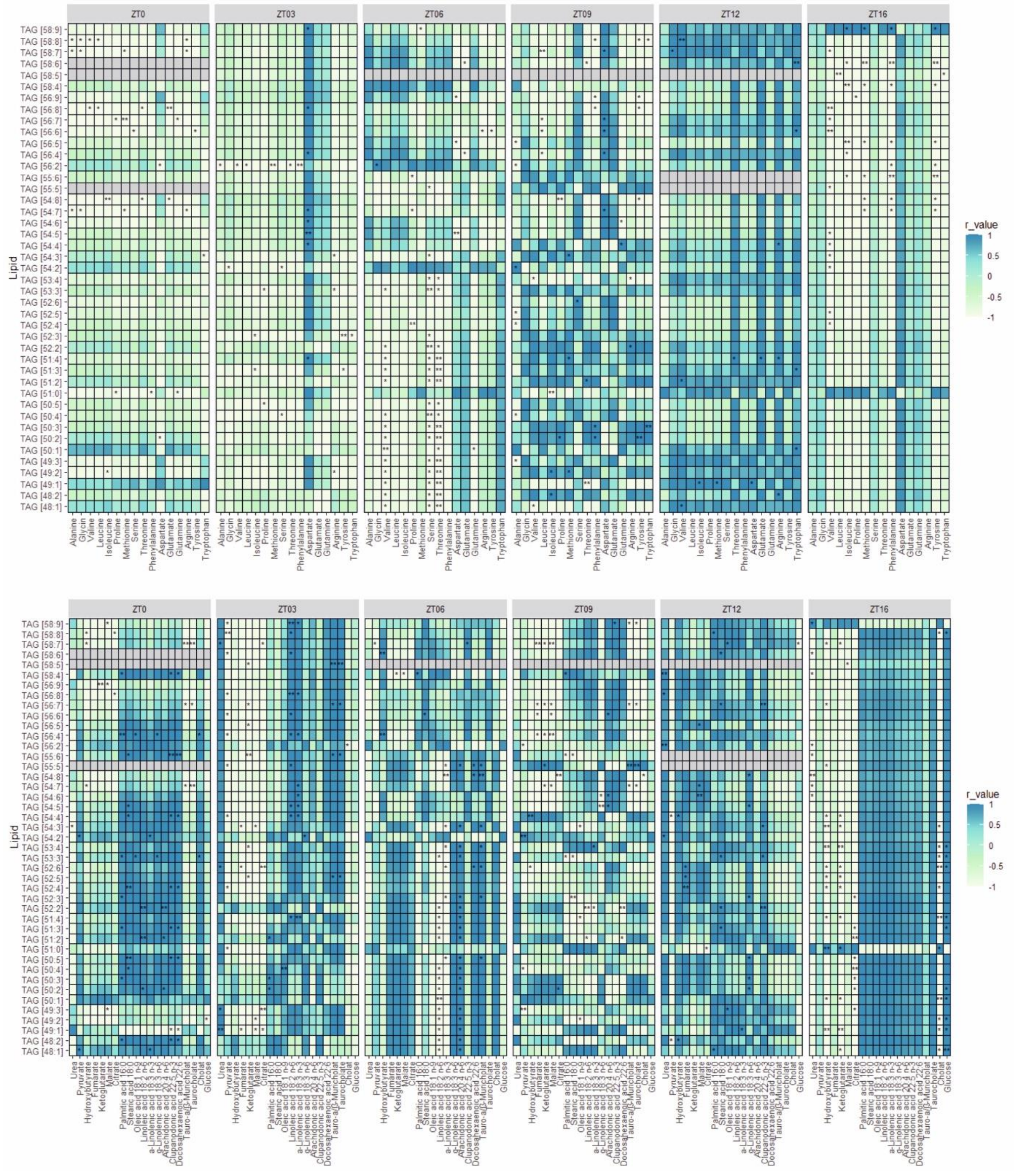

Fig. S2A. Correlation matrices of metabolome and lipids of the TAG species. Correlation matrices of metabolome data and lipids of the TAG species at time points ZT0, ZT6, ZT9, ZT12, and ZT16. The intensity of the colors indicates the degree of correlation between two parameters and reflects the strength of Pearson's correlation coefficient. Significance was tested using a Pearson correlation test and the asterisks display the p-values with *p < 0.05, and **p < 0.01.

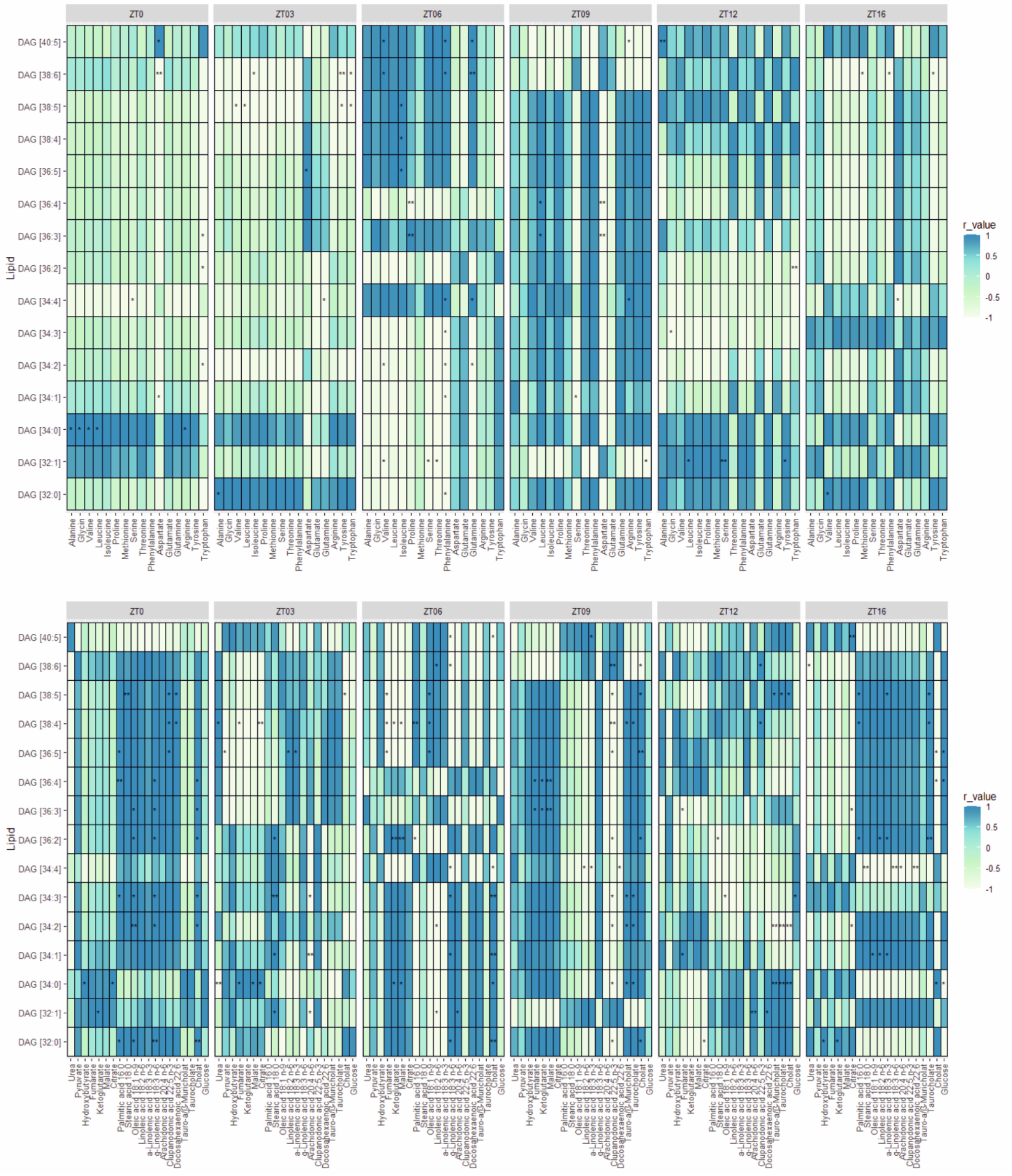

Fig. S2B. Correlation matrices of metabolome and lipids of the DAG species. Correlation matrices of metabolome data and lipids of the DAG species at time points ZT0, ZT6, ZT9, ZT12, and ZT16. The intensity of the colors indicates the degree of correlation between two parameters and reflects the strength of Pearson's correlation coefficient. Significance was tested using a Pearson correlation test and the asterisks display the p-values with $^*p < 0.05$, and $^{**}p < 0.01$.

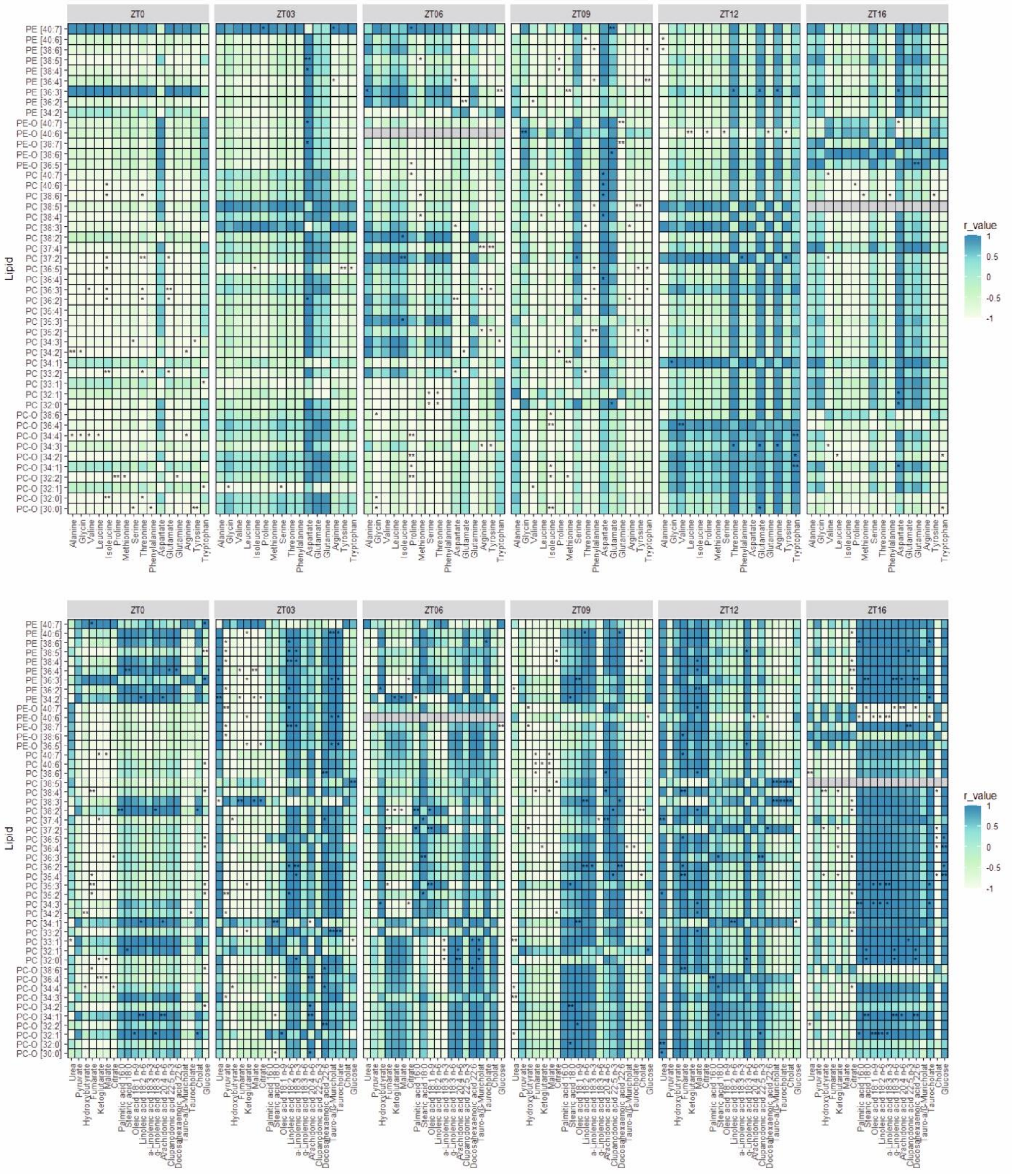

Fig. S2C. Correlation matrices of metabolome, phosphatidylcholines, phosphatidylethanolamines, phosphatidylcholine ether, and phosphatidylethanolamine ether. Correlation matrices of metabolome data and phosphatidylcholines, phosphatidylethanolamines, phosphatidylcholine ether and phosphatidylethanolamine ether at time points ZT0, ZT6, ZT9, ZT12, and ZT16. The intensity of the colors indicates the degree of correlation between two parameters and reflects the strength of Pearson's correlation coefficient. Significance was tested using a Pearson correlation test and the asterisks display the p-values with *p < 0.05, and **p < 0.01.

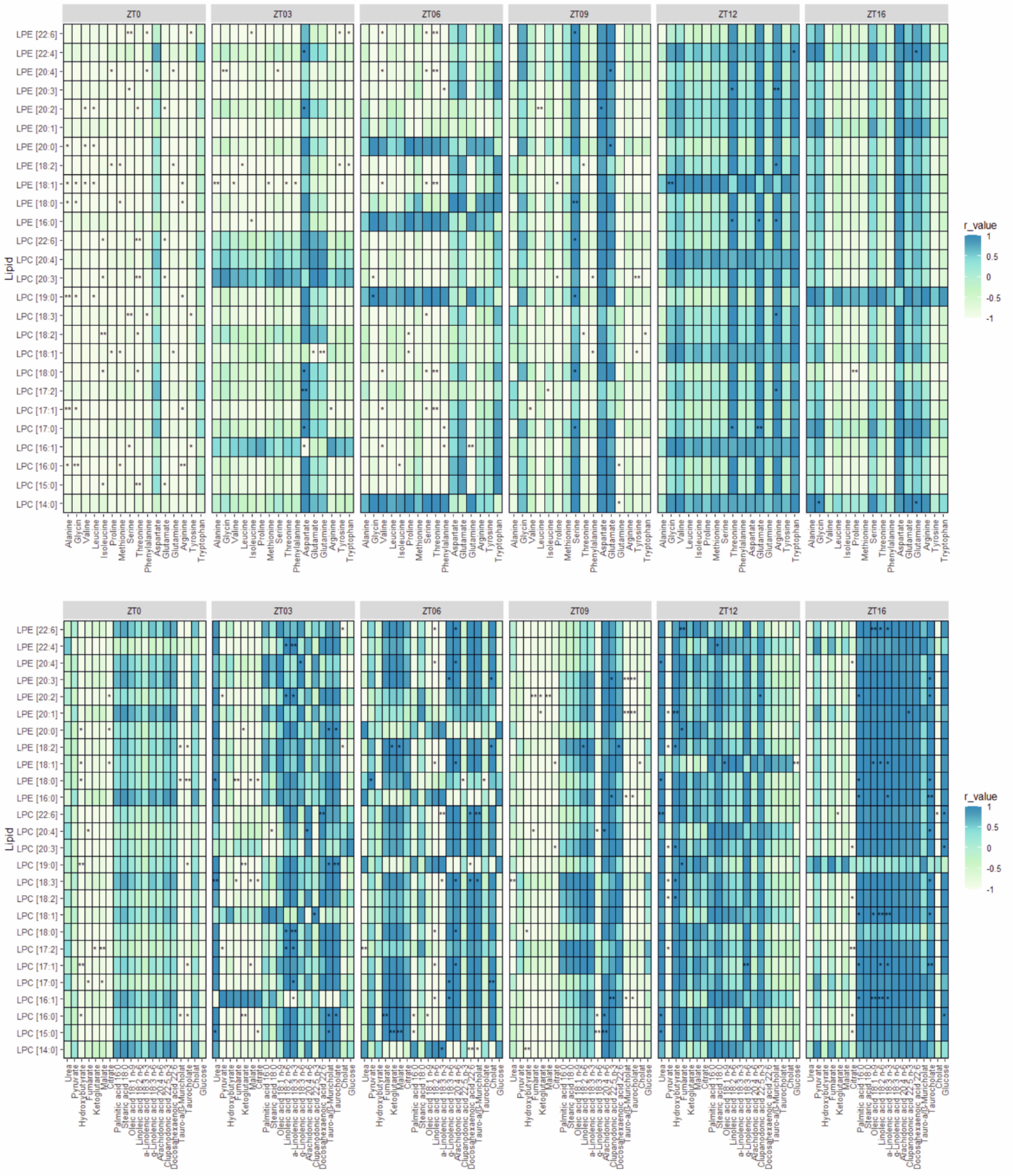

Fig. S2D. Correlation matrices of metabolome, lysophosphatidylcholines and lysophosphatidylethanolamines. Correlation matrices of metabolome data and lysophosphatidylcholines and lysophosphatidylethanolamines at time points ZT0, ZT6, ZT9, ZT12, and ZT16. The intensity of the colors indicates the degree of correlation between two parameters and reflects the strength of Pearson's correlation coefficient. Significance was tested using a Pearson correlation test and the asterisks display the p-values with $^*p < 0.05$, and $^{**}p < 0.01$.

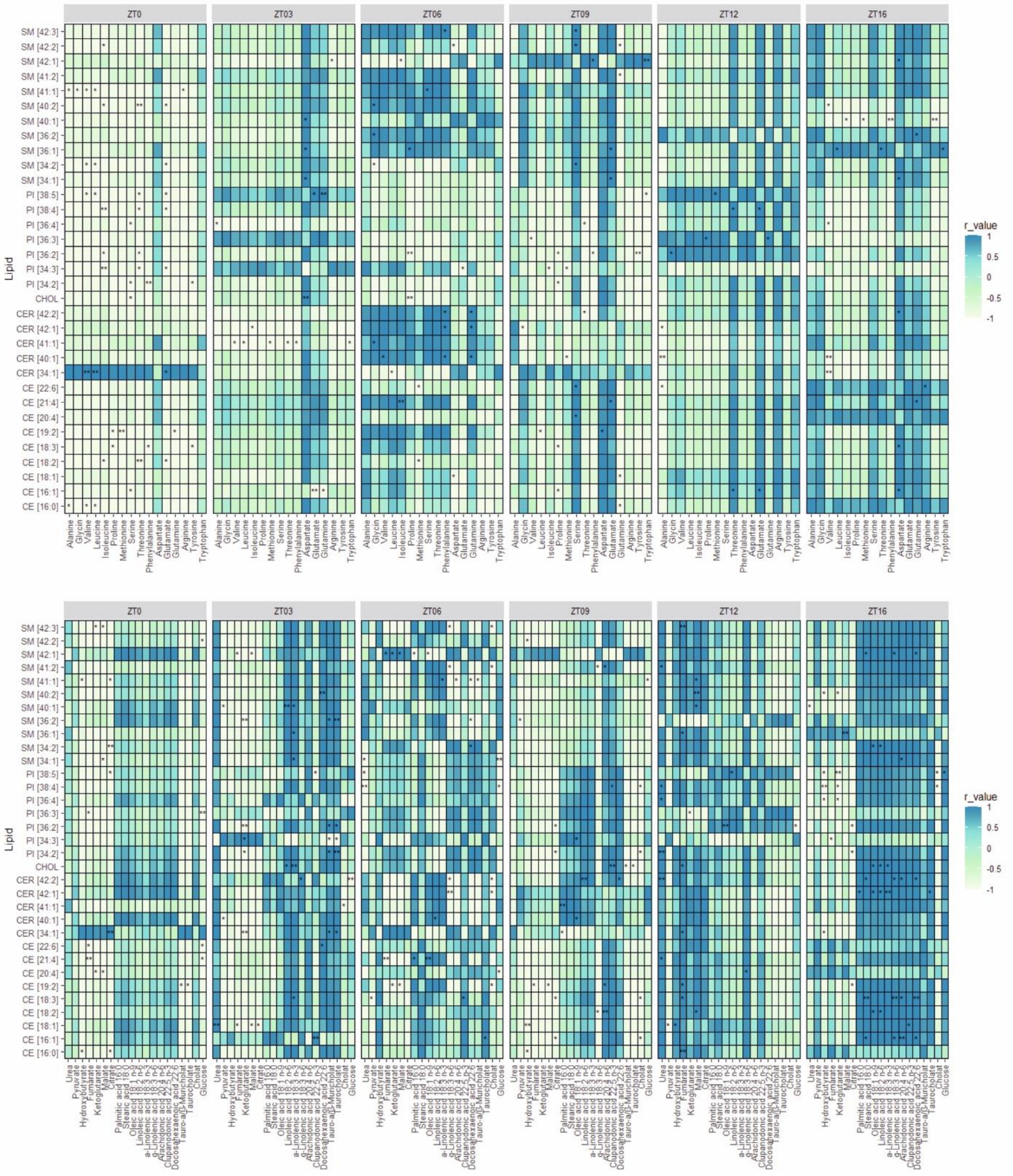

Fig. S2E. Correlation matrices of metabolome, cholesterolester, sphingomyelin, phosphatidylinositol, ceramides and cholesterol. Correlation matrices of metabolome data and cholesterolester, sphingomyelin, phosphatidyl-inositol, ceramides and cholesterol at time points ZT0, ZT6, ZT9, ZT12, and ZT16. The intensity of the colors indicates the degree of correlation between two parameters and reflects the strength of Pearson's correlation coefficient. Significance was tested using a Pearson correlation test and the asterisks display the p-values with *$p < 0.05$, and **$p < 0.001$.

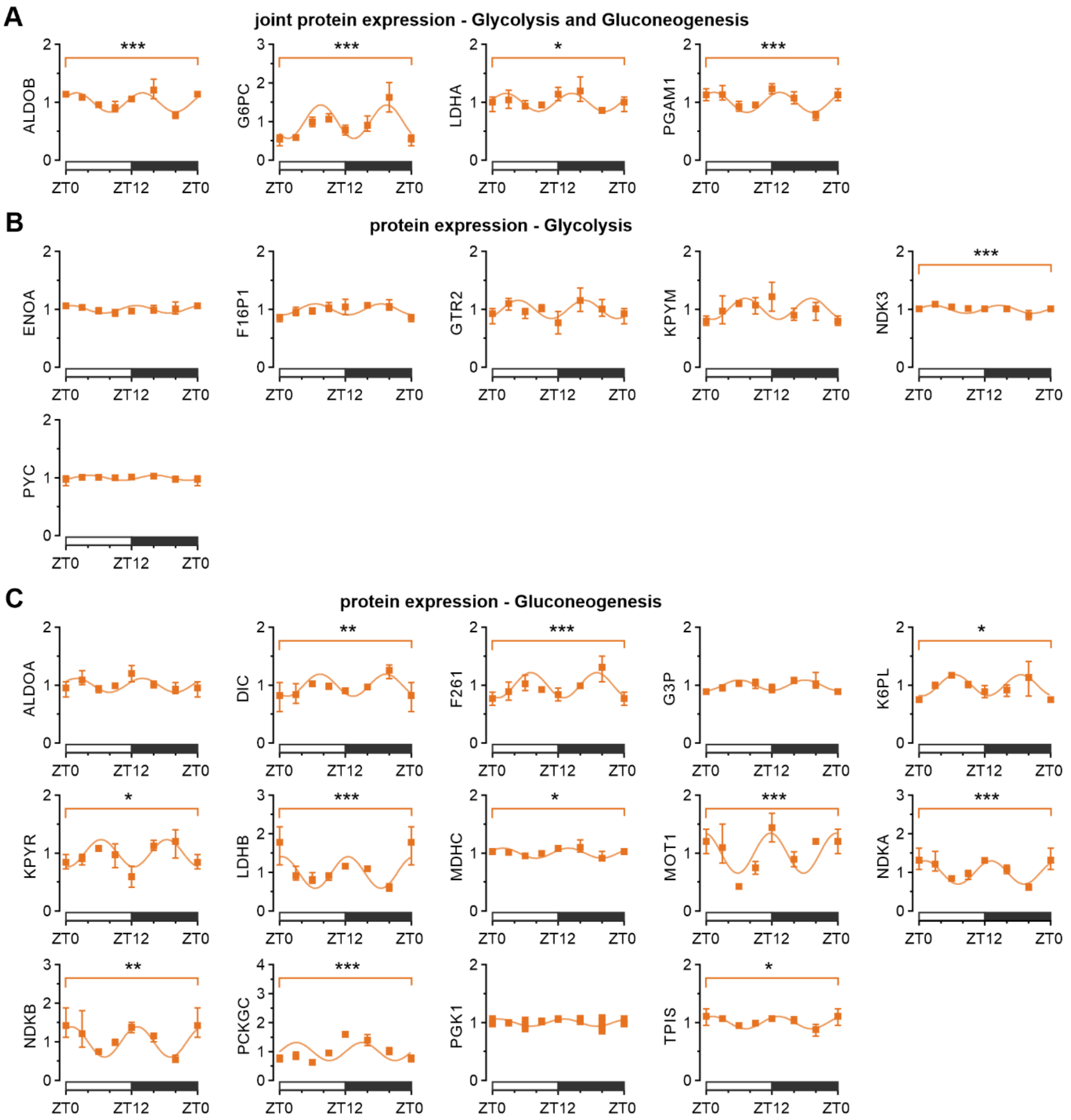

Fig. S3. Rhythmic variations of proteins of glycolysis and gluconeogenesis. Rhythmic regulation of significantly associated proteins, which occur (A) jointly in glycolysis and gluconeogenesis, (B) in glycolysis, and (C) in gluconeogenesis. Protein abundance is plotted in orange (n = 2 technical and N = 3-5 biological replicates). The solid lines depict best-fit sinusoidal wave functions with a 12-hour period. One-way ANOVA (protein abundance) with *$p < 0.05$, **$p < 0.01$, and ***$p < 0.001$.

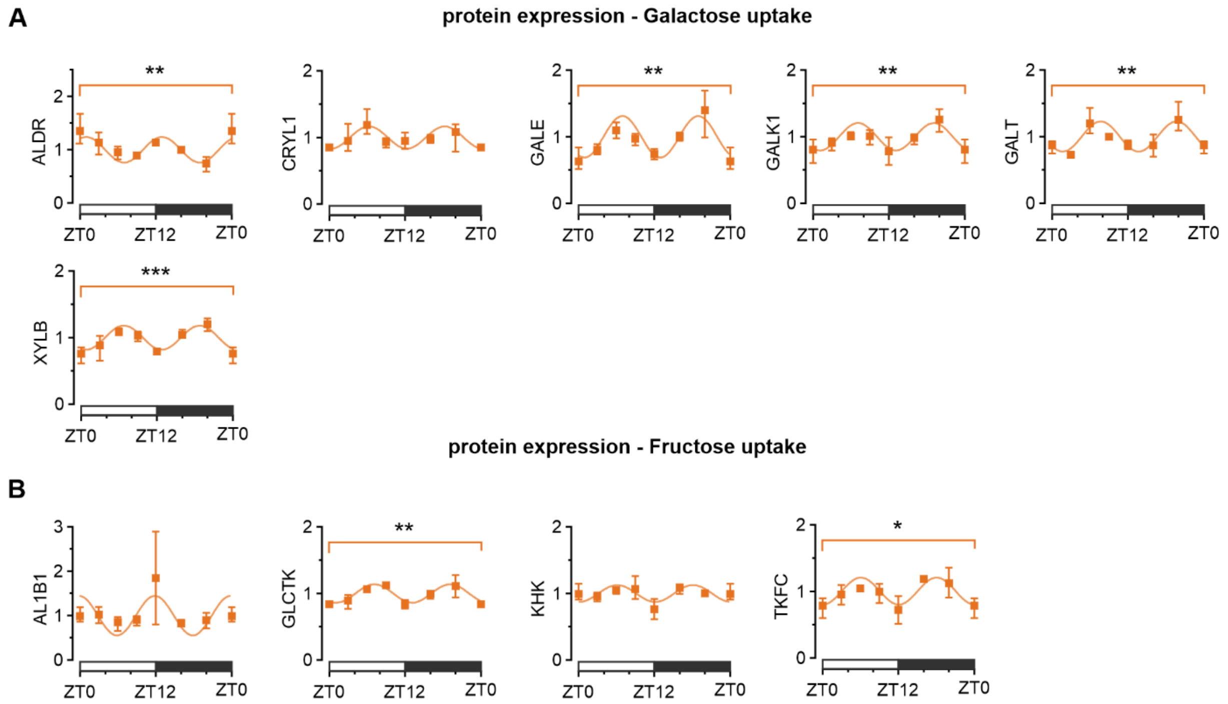

Fig. S4. Rhythmic variations of proteins of galactose and fructose uptake. Rhythmic regulation of significantly associated proteins, which occur in (A) galactose and (B) fructose uptake. Protein abundance is plotted in orange (n = 2 technical and N = 3-5 biological replicates. The solid lines depict best-fit sinusoidal wave functions with a 12-hour period. One-way ANOVA (protein abundance) with $*p < 0.05$, and $**p < 0.01$.

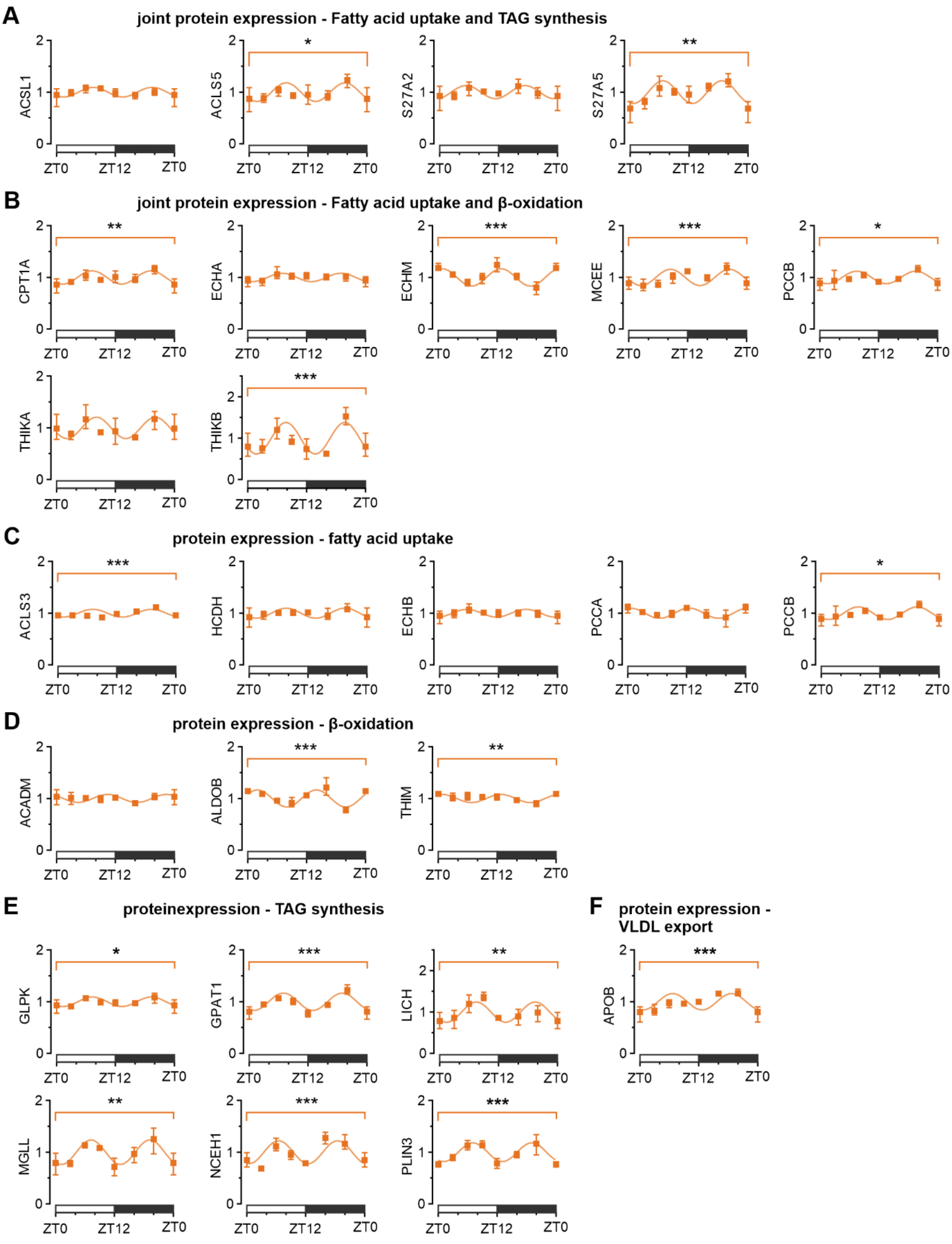

Fig. S5. Rhythmic variations of proteins of fatty acid uptake, TAG synthesis, VLDL export, and β-oxidation. Rhythmic regulation of significantly associated proteins, which occur (A) jointly in fatty acid uptake and TAG synthesis, (B) jointly in fatty acid uptake and β-oxidation, (C) in fatty acid uptake, (D) in β-oxidation, (E) in TAG synthesis, and (F) in VLDL export. Protein abundance is plotted in orange (n = 2 technical and N = 3-5 biological replicates). The solid lines depict best-fit sinusoidal wave functions with a 12-hour period. One-way ANOVA (protein abundance) with *p < 0.05, **p < 0.01, and ***p < 0.001.

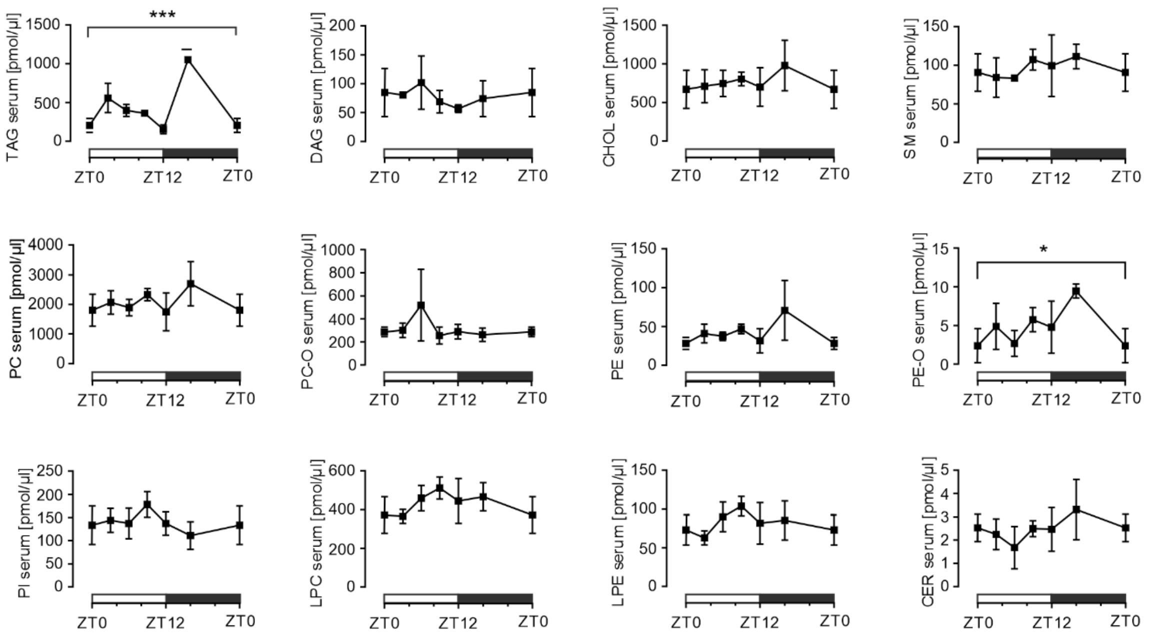

Fig. S6. Rhythmic lipidome profiles of the serum. Lipidome quantification of serum samples (n = 2 technical and N = 3 biological replicates). Values are plotted as mean ± standard deviation, one-way ANOVA with *p < 0.05, and ***p < 0.001.

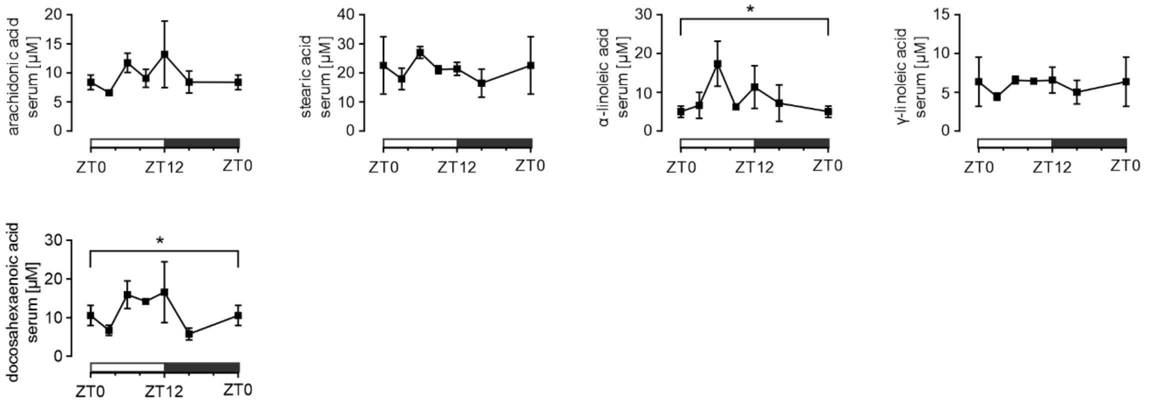

Fig. S7. Rhythmic profiles of serum fatty acids. Fatty acid quantification of serum samples (n = 2 technical and N = 3 biological replicates). Values are plotted as mean ± standard deviation, one-way ANOVA with *p < 0.05.

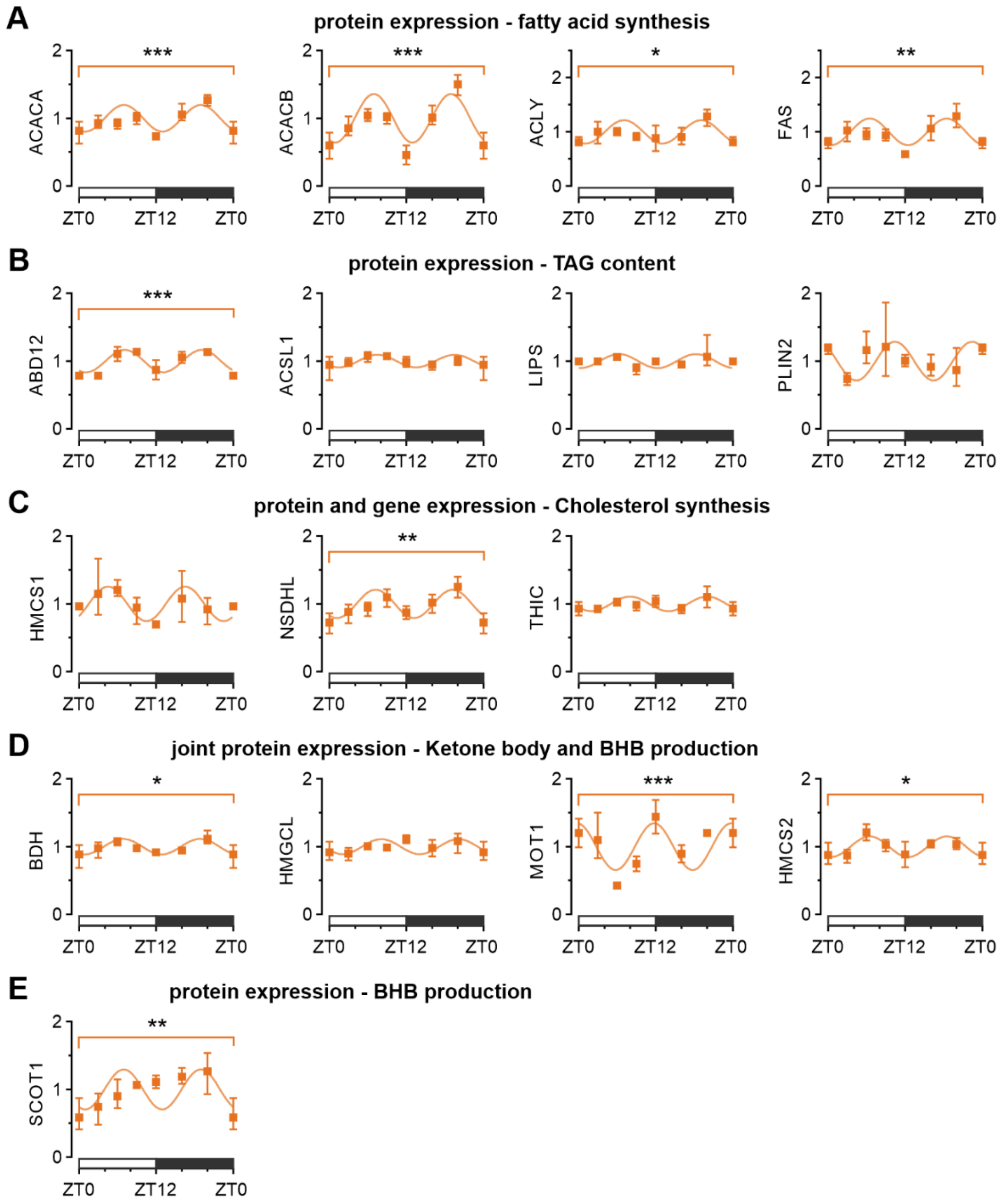

Fig. S8. Rhythmic variations of proteins of fatty acid synthesis, TAG content, cholesterol synthesis, ketone body, and β-hydroxybutyrate (BHB) production. Rhythmic regulation of significantly associated proteins, which occur in (A) fatty acid synthesis, (B) TAG content, (C) cholesterol synthesis, (D) jointly in ketone body and BHB production, and (E) BHB production. Protein abundance is plotted in orange (n = 2 technical and N = 3-5 biological replicates). The solid lines depict best-fit sinusoidal wave functions with a 12-hour period. One-way ANOVA (protein abundance) with *$p < 0.05$, **$p < 0.01$, and ***$p < 0.001$.

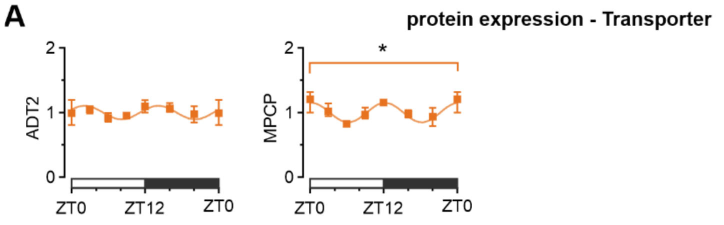
A
protein expression - Transporter
ADT2
MPCP
*
ZT0
ZT12
ZT0

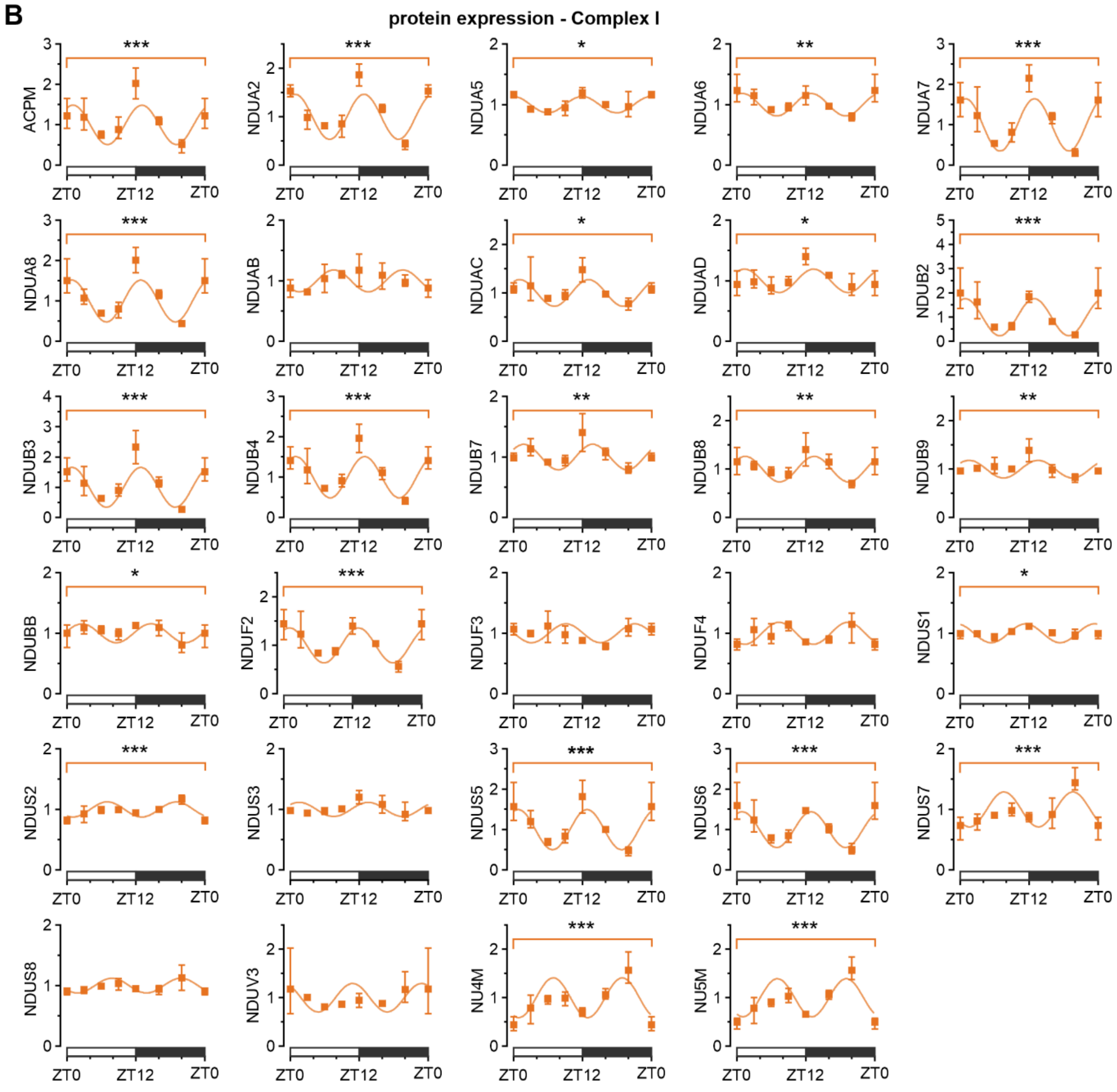
B
protein expression - Complex I
ACPM
NDUA2
NDUA5
NDUA6
NDUA7
NDUA8
NDUAB
NDUAC
NDUAD
NDUB2
NDUB3
NDUB4
NDUB7
NDUB8
NDUB9
NDUBB
NDUF2
NDUF3
NDUF4
NDUS1
NDUS2
NDUS3
NDUS5
NDUS6
NDUS7
NDUS8
NDUV3
NU4M
NU5M
ZT0
ZT12
ZT0

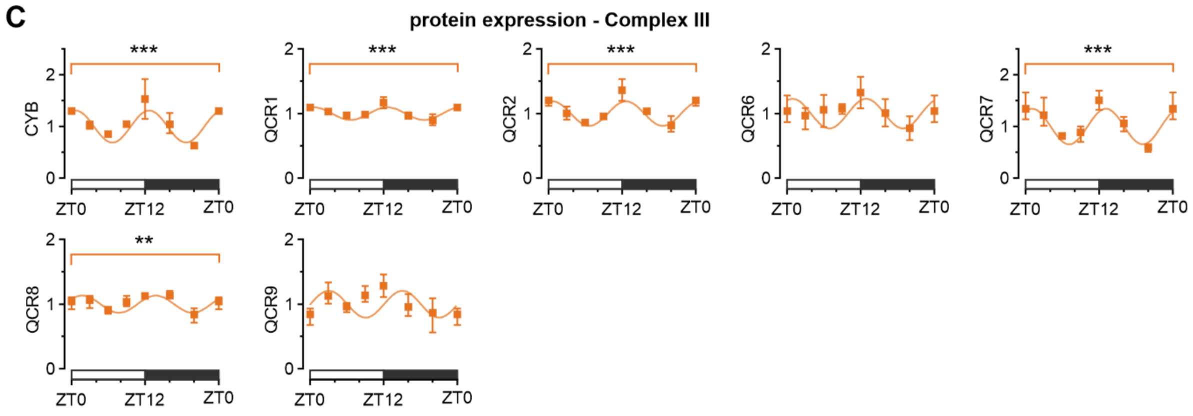
C
protein expression - Complex III
CYB
QCR1
QCR2
QCR6
QCR7
QCR8
QCR9
ZT0
ZT12
ZT0

D (continued)

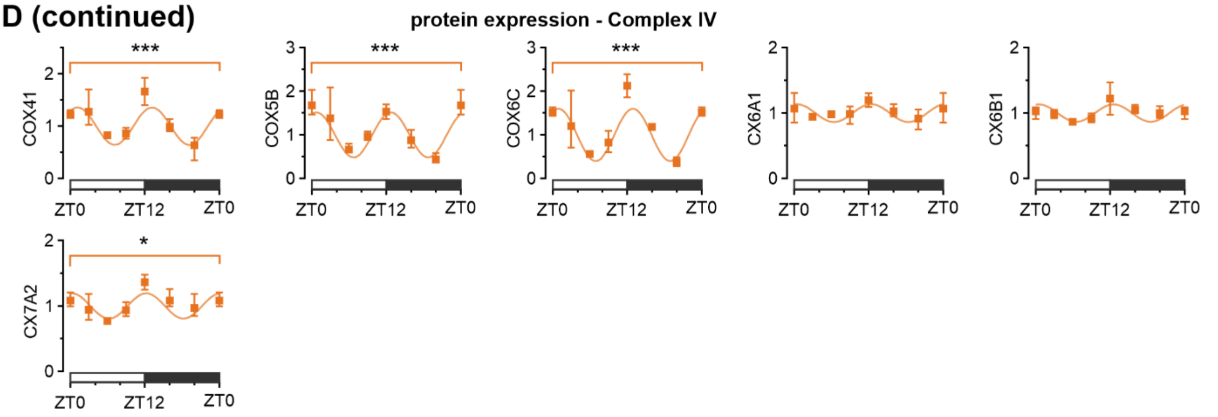

E

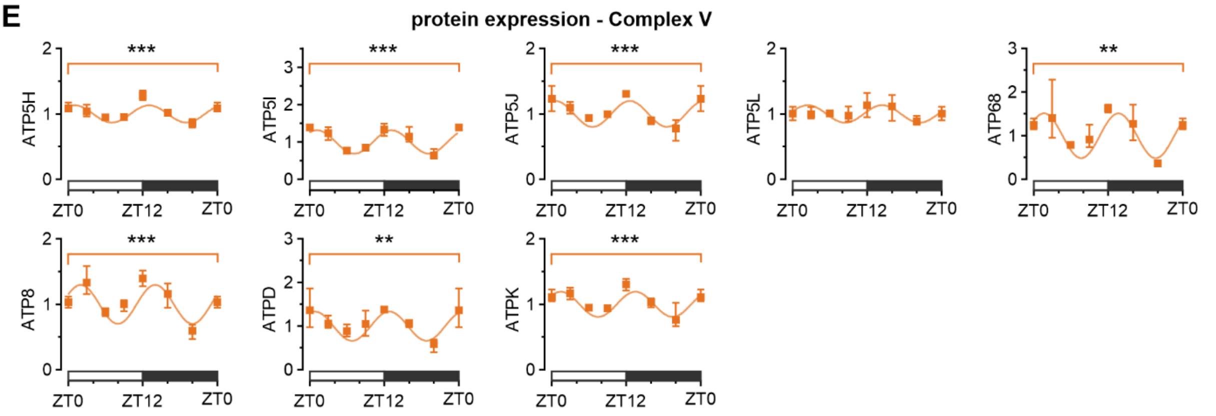

F

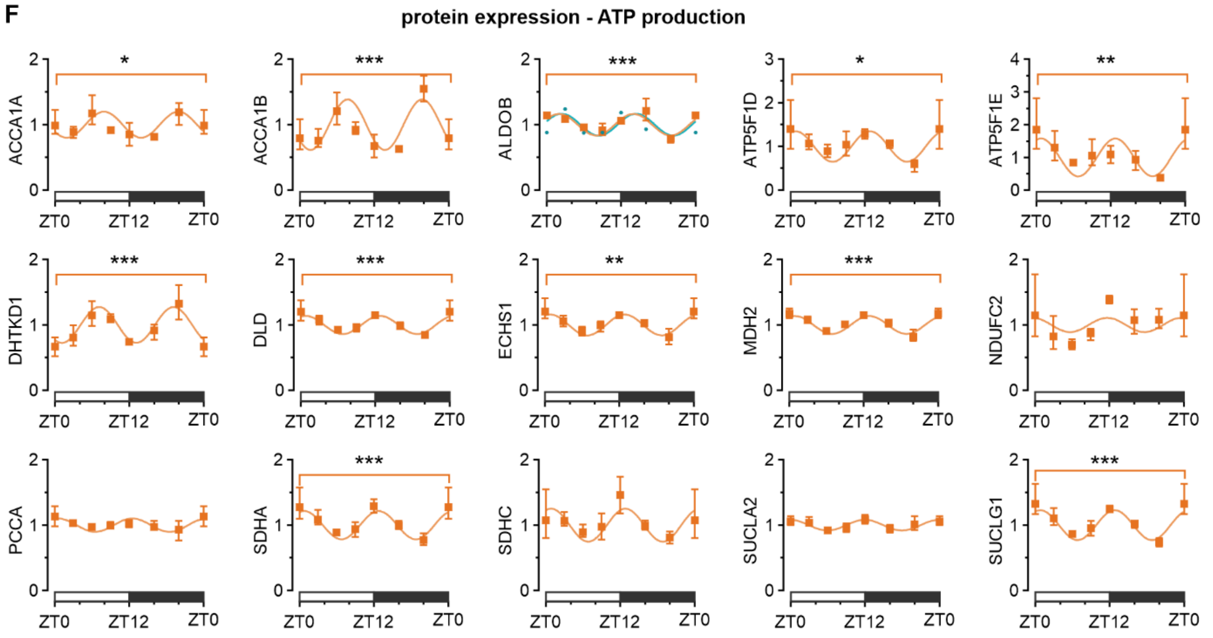

Fig. S9. Rhythmic variations of proteins of oxidative phosphorylation and ATP production.
Rhythmic regulation of significantly associated proteins of (A) transporters, (B) complex I, (C) complex III, (D) complex IV, (E) complex V of oxidative phosphorylation (F) proteins ATP production, which are not included in A-E. Protein abundance is plotted in orange (n = 2 technical and N = 3-5 biological replicates). The solid lines depict best-fit sinusoidal wave functions with a 12-hour period. One-way ANOVA (protein abundance) with *p < 0.05, **p < 0.01, and ***p < 0.001.

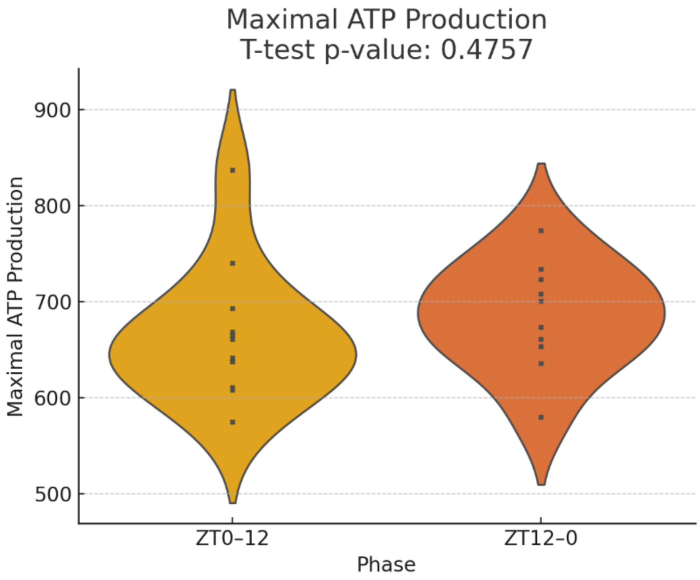

Fig. S10. Modeled hepatic ATP production capacity during light and dark phases.

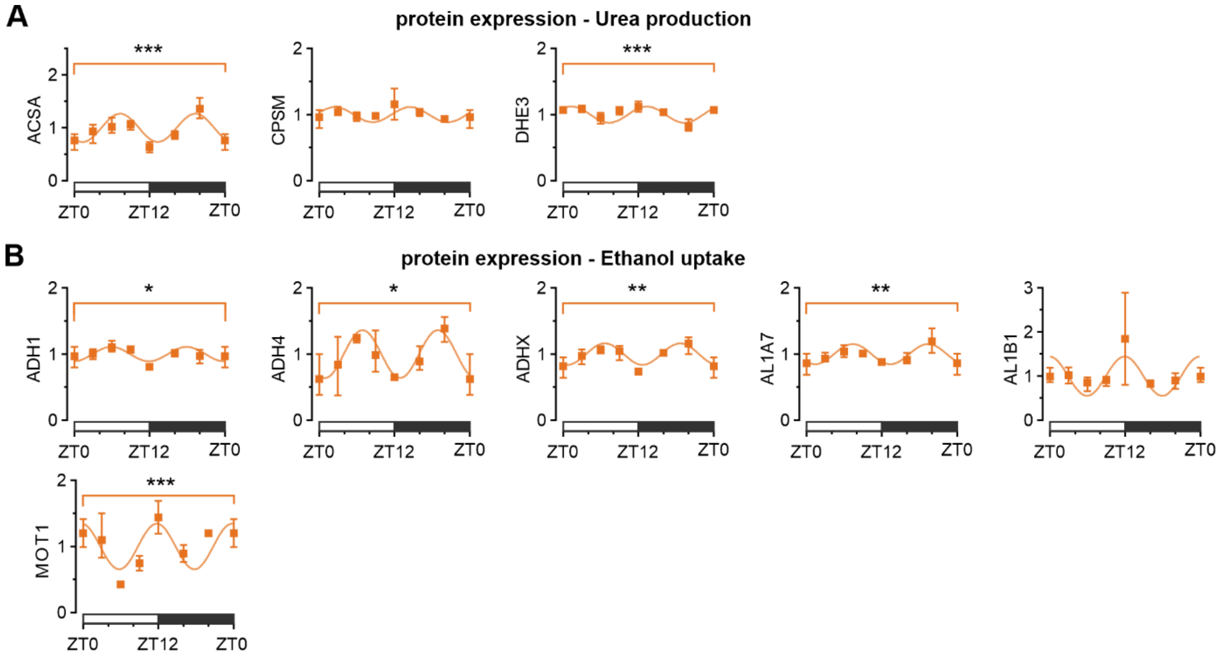

Fig. S11. Rhythmic variations of proteins of detoxification. Rhythmic regulation of significantly associated proteins of (A) urea production, and (B) ethanol metabolism. Protein abundance is plotted in orange (n = 2 technical and N = 3-5 biological replicates). The solid lines depict best-fit sinusoidal wave functions with a 12-hour period. One-way ANOVA (protein abundance) with *p < 0.05, **p < 0.01, and ***p < 0.001.

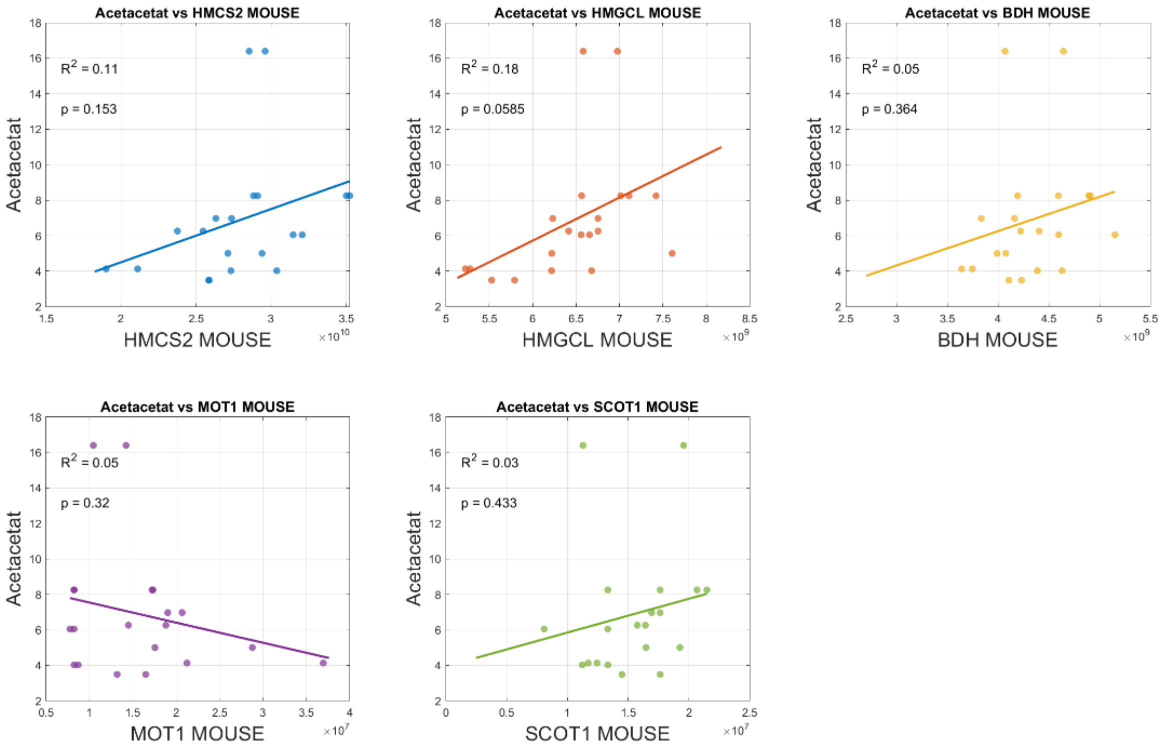

Fig. S12. Linear regression plots of serum ACAC versus protein levels.

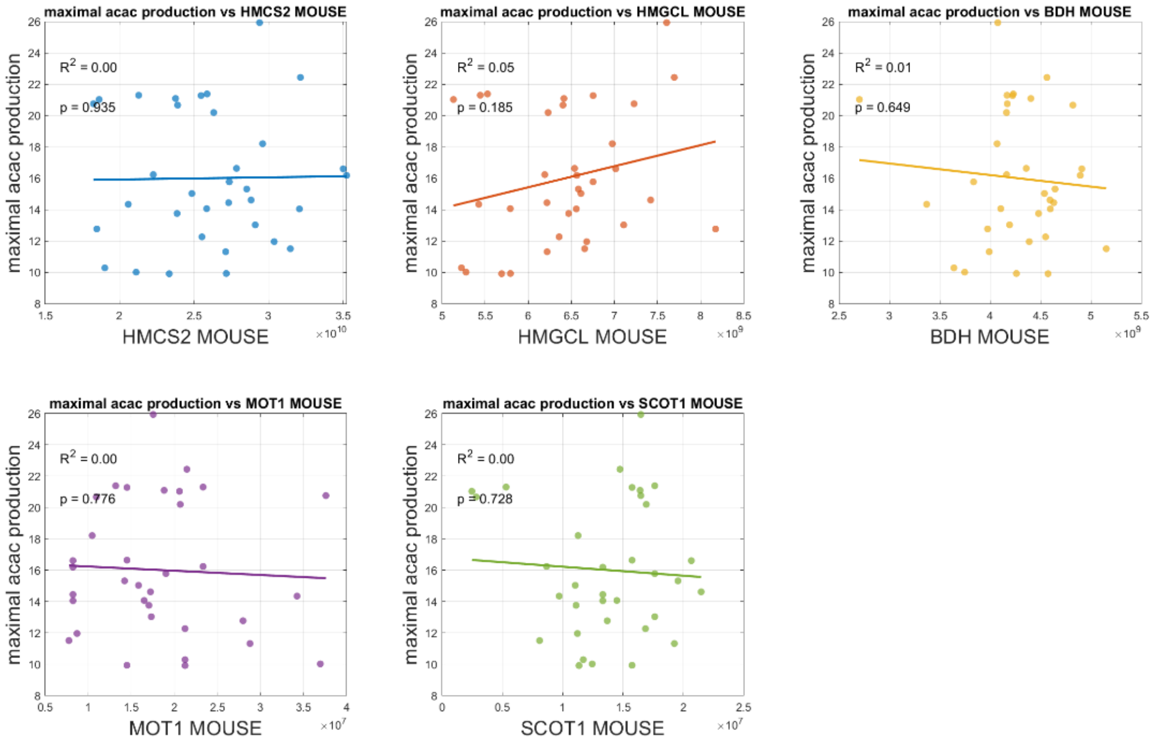

Fig. S13. Linear regression plots of modeled ACAC capacity versus protein levels.

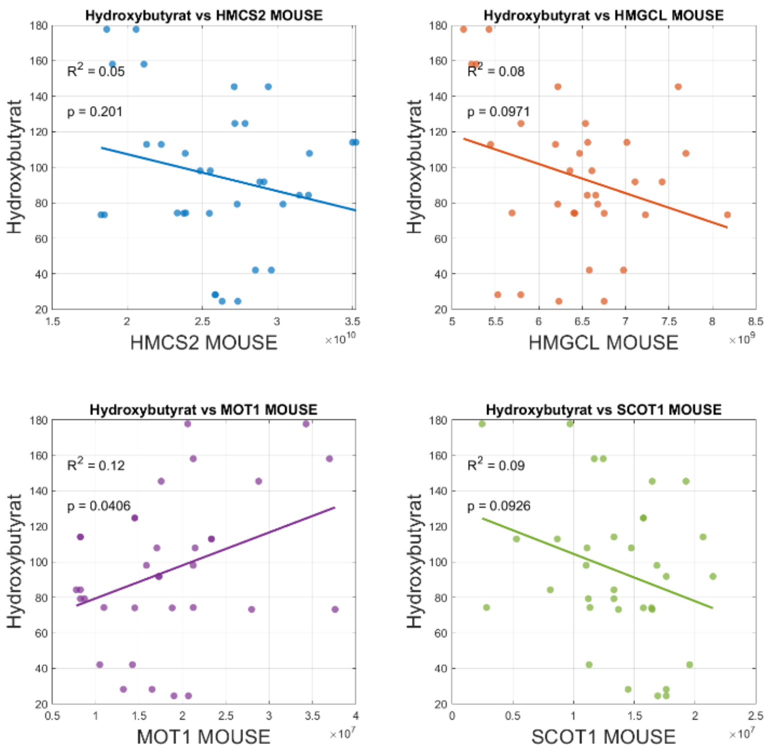

Fig. S14. Linear regression plots of serum BHB concentration versus protein levels.

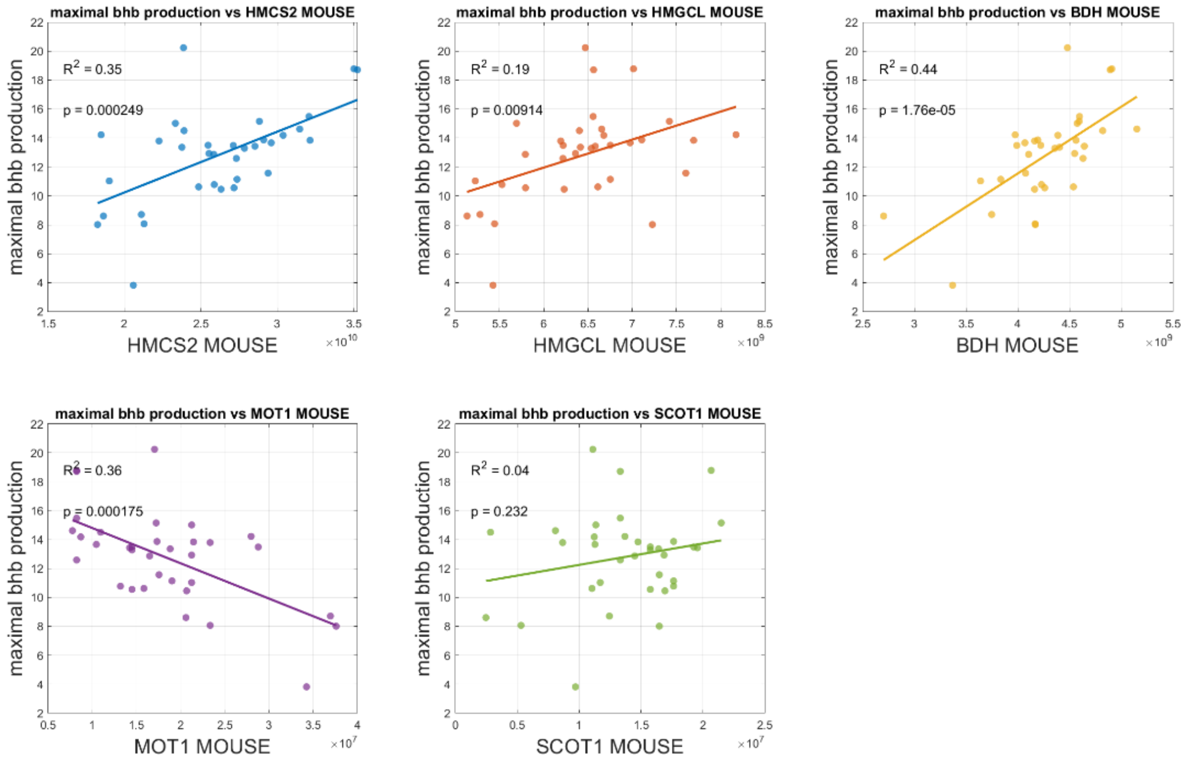

Fig. S15. Linear regression plots of modeled BHB capacity versus protein levels.

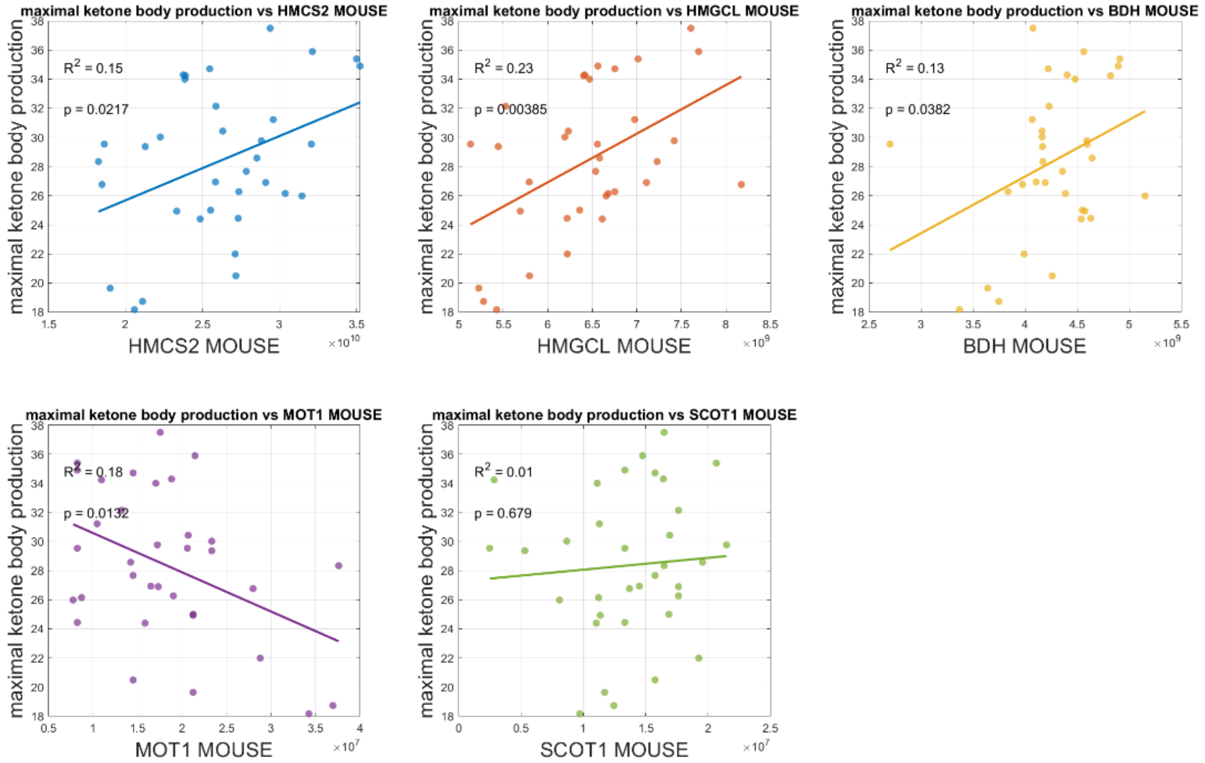

Fig. S16. Linear regression plots of modeled total ketone body production capacity versus protein levels.

SUPPLEMENTARY MATERIAL

SUPPLEMENTARY TABLES

Table S1. Proteome data. (see ESM_2)

Table S2. Lipidome data. (see ESM_3)

Table S3. Metabolome data. (see ESM_4)

Table S4. Metabolic functions (within physiological range or maximal capacity) and all significantly associated proteins with their p-values. (see ESM_5)

Table S5. Comparative model fits for rhythmicity analysis of metabolic functions: constant vs. 12h and 24h sinusoidal models. (see ESM_6)

Table S6. Biodare2 Analysis of all OMIC data. (see ESM_7)

Table S7. Pearson's correlation coefficients and corresponding p-values for ZT0-ZT16. (see ESM_8)

Table S8. P-values of correlation of maximal capacity of metabolic functions within physiological ranges with metabolites detected in serum.

Metabolite / Function	Glucose	Pyruvate
Gluconeogenesis	0.078	0.012

Table S9. P-values of correlation of maximal capacity of metabolic functions within physiological ranges with metabolites detected in serum.

Metabolite / Function	Palmitic acid	Oleic acid	Linoleic acid	Clupano-donic acid
Fatty acid uptake	3.35E-03	1.32E-03	3,67E-04	1.57E-02
TAG synthesis	6.36E-05	8.36E-06	4,87E-06	1.04E-03
TAG content	4.81E-02	2.92E-02	4,06E-02	1.24E-02
VLDL export	1.07E-03	4.41E-04	7,20E-05	1.36E-02

Table S10. P-values of correlation of maximal capacity of metabolic functions within physiological ranges with metabolites detected in serum.

Metabolite / Function	Stearic acid	α-linolenic acid	γ-linolenic acid	Arachidonic acid	Docosahexa-enoic acid
Fatty acid uptake	1.58E-01	2.57E-04	3.33E-01	1.68E-02	1.18E-01
TAG synthesis	3.86E-02	4.15E-06	3.15E-01	1.01E-02	3.46E-02
TAG content	6.10E-02	8.85E-02	3.25E-01	1.07E-01	1.37E-02
VLDL export	1.13E-01	6.24E-05	1.71E-01	3.74E-03	1.06E-01

n.s. – not significant

Table S11. Mitochondrial proteins selected via MitoCharta 3.0. (see ESM_9)

Table S12. Z-Score from IPA analysis of mitochondrial functions

Miotochondrial function	ZT3/ZT0	ZT6/ZT0	ZT9/ZT0	ZT12/ZT0	ZT16/ZT0	ZT20/ZT0
electron transport /ATP synth.	-1.633	-1.361	-0.816	4.082	-0.272	-2.335
oxidative phosphorylation	-0.944	-1.483	-1.214	3.101	0.405	-2.177
mito. dysfunction	1	1.5	1.25	-2.75	-0.25	1.89
mito. fatty acid beta-oxidation	-1.732	-1.155	0	-0.577	-0.577	0
mito. ptrotein degradation	-0.169	-1.183	-0.169	0.845	0.845	-0.507
mito. protein import	0.707	0.707	0	0.707	0.707	0.707

Table S13. P-values of correlation of maximal capacity of metabolic functions within physiological ranges with metabolites detected in serum.

Metabolite / Function	Aspartate	Glutamate	Urea
Urea production	5.67E-01	6.95E-01	8.16E-01

n.s. – not significant

SUPPLEMENTARY REFERENCES

1. Folch J, Lees M, Sloane Stanley GH, *A simple method for the isolation and purification of total lipides from animal tissues.* J Biol Chem, (1957). **226**(1): p. 497-509. DOI: 10.1016/S0021-9258(18)64849-5 M4 - Citavi.
2. Schuhmann K, Almeida R, Baumert M, Herzog R, Bornstein SR, Shevchenko A, *Shotgun lipidomics on a LTQ Orbitrap mass spectrometer by successive switching between acquisition polarity modes.* J Mass Spectrom, (2012). **47**(1): p. 96-104. DOI: 10.1002/jms.2031.
3. Liebisch G, Binder M, Schifferer R, Langmann T, Schulz B, Schmitz G, *High throughput quantification of cholesterol and cholesteryl ester by electrospray ionization tandem mass spectrometry (ESI-MS/MS).* Biochim Biophys Acta, (2006). **1761**(1): p. 121-8. DOI: 10.1016/j.bbalip.2005.12.007.
4. Herzog R, Schwudke D, Schuhmann K, Sampaio JL, Bornstein SR, Schroeder M, Shevchenko A, *A novel informatics concept for high-throughput shotgun lipidomics based on the molecular fragmentation query language.* Genome Biol, (2011). **12**(1): p. R8. DOI: 10.1186/gb-2011-12-1-r8.
5. Hofmann U, Maier K, Niebel A, Vacun G, Reuss M, Mauch K, *Identification of metabolic fluxes in hepatic cells from transient 13C-labeling experiments: Part I. Experimental observations.* Biotechnol Bioeng, (2008). **100**(2): p. 344-54. DOI: 10.1002/bit.21747.
6. Maier K, Hofmann U, Reuss M, Mauch K, *Dynamics and control of the central carbon metabolism in hepatoma cells.* BMC Syst Biol, (2010). **4**: p. 54. DOI: 10.1186/1752-0509-4-54.
7. Berndt N, Bulik S, Wallach I, Wunsch T, Konig M, Stockmann M, Meierhofer D, Holzhutter HG, *HEPATOKIN1 is a biochemistry-based model of liver metabolism for applications in medicine and pharmacology.* Nat Commun, (2018). **9**(1): p. 2386. DOI: 10.1038/s41467-018-04720-9.

8. Berndt N, Hudert CA, Eckstein J, Loddenkemper C, Henning S, Bufler P, Meierhofer D, Sack I, Wiegand S, Wallach I, Holzhutter HG, *Alterations of Central Liver Metabolism of Pediatric Patients with Non-Alcoholic Fatty Liver Disease.* Int J Mol Sci, (2022). **23**(19). DOI: 10.3390/ijms231911072.
9. Berndt N, Kann O, Holzhutter HG, *Physiology-based kinetic modeling of neuronal energy metabolism unravels the molecular basis of NAD(P)H fluorescence transients.* J Cereb Blood Flow Metab, (2015). **35**(9): p. 1494-506. DOI: 10.1038/jcbfm.2015.70.
10. Bulik S, Holzhutter HG, Berndt N, *The relative importance of kinetic mechanisms and variable enzyme abundances for the regulation of hepatic glucose metabolism--insights from mathematical modeling.* BMC Biol, (2016). **14**: p. 15. DOI: 10.1186/s12915-016-0237-6.
11. Wallstab C, Eleftheriadou D, Schulz T, Damm G, Seehofer D, Borlak J, Holzhutter HG, Berndt N, *A unifying mathematical model of lipid droplet metabolism reveals key molecular players in the development of hepatic steatosis.* FEBS J, (2017). **284**(19): p. 3245-3261. DOI: 10.1111/febs.14189.
12. Berndt N, Eckstein J, Wallach I, Nordmeyer S, Kelm M, Kirchner M, Goubergrits L, Schafstedde M, Hennemuth A, Kraus M, Grune T, Mertins P, Kuehne T, Holzhutter HG, *CARDIOKIN1: Computational Assessment of Myocardial Metabolic Capability in Healthy Controls and Patients With Valve Diseases.* Circulation, (2021). **144**(24): p. 1926-1939. DOI: 10.1161/CIRCULATIONAHA.121.055646.
